# EpiSegMix: a flexible distribution hidden Markov model with duration modeling for chromatin state discovery

**DOI:** 10.1093/bioinformatics/btae178

**Published:** 2024-04-02

**Authors:** Johanna Elena Schmitz, Nihit Aggarwal, Lukas Laufer, Jörn Walter, Abdulrahman Salhab, Sven Rahmann

**Affiliations:** Algorithmic Bioinformatics, Center for Bioinformatics Saar, Saarland Informatics Campus, 66123 Saarbrücken, Germany; Fakultät MI, Saarland University, Saarland Informatics Campus, 66123 Saarbrücken, Germany; Saarbrücken Graduate School of Computer Science, Saarland Informatics Campus, 66123 Saarbrücken, Germany; Department of Genetics, Saarland University, 66123 Saarbrücken, Germany; Department of Genetics, Saarland University, 66123 Saarbrücken, Germany; Department of Genetics, Saarland University, 66123 Saarbrücken, Germany; Department of Genetics, Saarland University, 66123 Saarbrücken, Germany; Genomics Data Science Core, Integrated Genomics Services, Sidra Medicine, Doha, Qatar; Algorithmic Bioinformatics, Center for Bioinformatics Saar, Saarland Informatics Campus, 66123 Saarbrücken, Germany; Fakultät MI, Saarland University, Saarland Informatics Campus, 66123 Saarbrücken, Germany

## Abstract

**Motivation:**

Automated chromatin segmentation based on ChIP-seq (chromatin immunoprecipitation followed by sequencing) data reveals insights into the epigenetic regulation of chromatin accessibility. Existing segmentation methods are constrained by simplifying modeling assumptions, which may have a negative impact on the segmentation quality.

**Results:**

We introduce EpiSegMix, a novel segmentation method based on a hidden Markov model with flexible read count distribution types and state duration modeling, allowing for a more flexible modeling of both histone signals and segment lengths. In a comparison with existing tools, ChromHMM, Segway, and EpiCSeg, we show that EpiSegMix is more predictive of cell biology, such as gene expression. Its flexible framework enables it to fit an accurate probabilistic model, which has the potential to increase the biological interpretability of chromatin states.

**Availability and implementation:**

Source code: https://gitlab.com/rahmannlab/episegmix.

## 1 Introduction

Each cell in a eukaryotic organism contains the same genetic information to build all required structural and functional gene products. However, cell-to-cell variation is essential for having specialized tissues with distinct physiological functions and to adapt to environmental changes (Cavalli and Heard 2019, Carter and Zhao 2021). This necessitates an additional layer of processes regulating gene expression to enable cell differentiation and to maintain cellular identities throughout cell divisions (Allis and Jenuwein 2016). Among the mechanisms tightly regulating gene expression are transcription factors and epigenetic modifications, like DNA methylation and histone modifications.

### 1.1 Histone modifications

With increasing knowledge about the role of histone modifications in altering the chromatin structure and DNA accessibility, it became apparent that different histone modifications are enriched in chromatin regions with distinct functional roles (Baker 2011). For example, modification H3K4me3 [in histone protein H3, the lysine at position 4 (K4) is trimethylated (me3)] is enriched in promoters and can be linked to transcriptional activation; H3K36me3 is enriched in active genes, and H3K27me3 can be associated with gene repression by the Polycomb protein complex (Blackledge and Klose 2021). Densely packed chromatin, called heterochromatin, is typically characterized by low levels of acetylation, whereas open, actively transcribed chromatin, called euchromatin, shows enrichment of acetylated lysine (Bannister and Kouzarides 2011). Combinatorial patterns of multiple histone modifications allow us to characterize so-called *chromatin states* that describe the different functional states of both coding and noncoding regions in the genome (Baker 2011).

### 1.2 Chromatin immunoprecipitation followed by sequencing

Chromatin immunoprecipitation followed by sequencing (ChIP-seq) enables the generation of genome-wide histone maps in high-throughput experiments (Barski *et al.* 2007). For ChIP-seq, DNA-binding proteins of interest, such as specifically modified histones or transcription factors, are tagged with specific antibodies. After chromatin shearing, DNA fragments bound to the desired proteins or protein-modifications are captured and the bound DNA is extracted for sequencing. Reads are mapped to the reference genome to infer the positioning of histone marks across the genome (Park 2009). The positional enrichment of reads is compromised by a certain amount of noise.

### 1.3 Probabilistic models for segmentation

**Figure 1.**
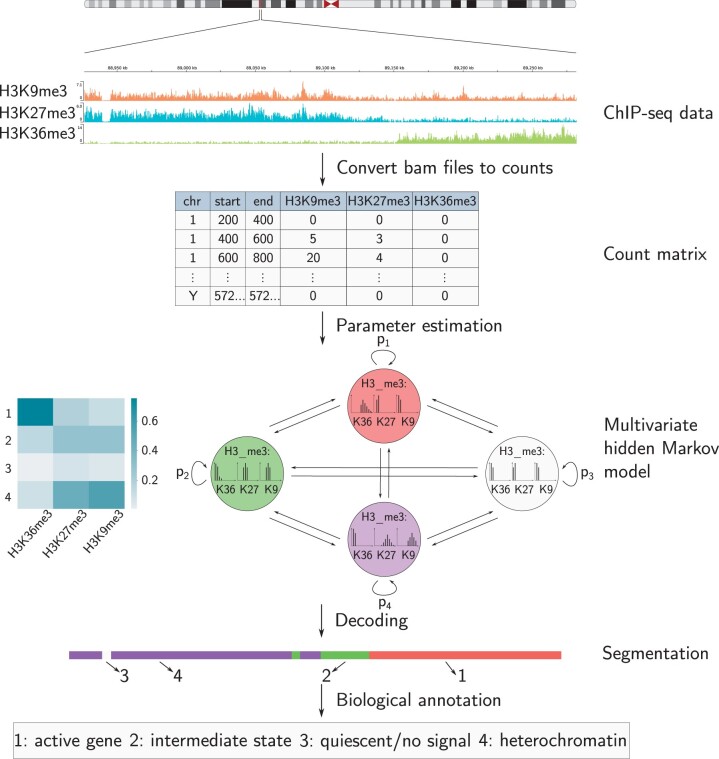
Chromatin segmentation: the reads of a ChIP-seq experiment are converted into a count matrix by counting the number of reads mapping to each nonoverlapping 200-bp genomic interval. The states of a multivariate HMM capture patterns in the multivariate read count distribution of the histone marks, and the transition probabilities between states capture the relations between adjacent chromatin states.

The availability of genome-wide ChIP-seq data led to the development of automated methods for genome segmentation and annotation. These methods use a probabilistic model to detect recurrent patterns of epigenetic marks using the aligned reads to determine the signal intensity at different positions in the genome (Fig. 1). HMMSeg (Day *et al.* 2007), ChromHMM (Ernst and Kellis 2010), and EpiCSeg (Mammana and Chung 2015) are based on hidden Markov models (HMMs), Segway, and Segway 2.0 (Chan *et al.* 2018) fit a Gaussian mixture model using a dynamic Bayesian network and Daneshpajouh *et al.* (2022) developed a state–space model assuming that the observed data is a linear function of the state-specific parameter matrix plus Gaussian noise. Marco *et al.* (2017) proposed a hierarchical HMM to simultaneously generate two segmentations at different length scales. The first (nucleosome level) segmentation works similarly to ChromHHM and captures histone modification patterns. The second (domain level) segmentation interprets sequences of nucleosome states (e.g. a super-enhancer domain state combining strong, weak, and flanking enhancer nucleosome states). In the present work, we focus on the accurate modeling of the nucleosome level. For a more comprehensive review, we refer to Libbrecht *et al.* (2021).

### 1.4 HMMs

Multivariate HMMs capture both combinatorial patterns of multiple histone marks and adjacency relations between different genomic elements, which makes them a prominent tool for chromatin state discovery (Lee and Park 2014). An HMM describes two stochastic processes, an invisible Markov chain consisting of a finite set of hidden states and a visible process of observable signals. Here, the hidden states correspond to chromatin states and the observable signals to the observed read count vector per genomic region. Each hidden state has state-specific probabilities of emitting an observation, called *emission probabilities*. For chromatin segmentation, the genome is divided into nonoverlapping intervals (of typical length 200 bp), such that each observation is a vector of counts corresponding to the number of reads assigned to the interval per histone mark. Thus, the emission probabilities of a single hidden state capture a specific combinatorial pattern of multiple histone marks. Different states may hence define different functional genomic elements, such as promoters, enhancers or gene bodies. In addition, the relations between adjacent chromatin states are modeled via transition probabilities, which determine, for each state, the probabilities to either stay in the same state or to transition to another state.

### 1.5 Modeling assumptions

The ability of an HMM to detect patterns that correspond to biologically meaningful chromatin states is constrained by the modeling assumptions underlying the emission and transition probabilities. These assumptions are thus a distinguishing feature of existing HMM-based methods. For example, ChromHMM fits an HMM on binarized data (high versus low read count), where the emission probabilities are assumed to be independent Bernoulli experiments, and EpiCSeg models the emission probabilities using a Negative Multinomial distribution. Previous analyses of ChIP-seq data have shown that the read count distributions in some states and for some histone marks may be overdispersed and skewed, partly caused by differential protection against sonication, unequal binding affinity of distinct antibodies, sequence dependent PCR amplification and discrepancies when mapping to repeat-rich regions, which all introduce bias to the data (Diaz *et al.* 2012). Not all observed combinations of overdispersion and skewness can be captured by the commonly used probability distribution families, such as the Negative Binomial distribution. Furthermore, Beacon *et al.* (2021) showed that histone marks that are enriched in short domains, like promoters or TSS, are typically characterized by narrow peaks with high signal intensities, while histone marks enriched in broad domains, like heterochromatic regions, have lower signal intensities. Thus, regions in the genome covered by the same chromatin state may have different lengths, e.g. short promoters and long heterochromatic regions. Existing tools only model a single duration distribution type (Geometric) with exponentially decreasing probabilities, and can only fit the mean length of a region, but not its shape to the observed data. Hyperparameters, such as introduced by Segway, allow to place a prior on the expected segment length, but are still prone to the above limitations due to the unchanged geometric distribution type (Hoffman *et al.* 2012). Furthermore, they are either limited to the same parameters for all states or can be set for each numeric label, which may require extensive manual fine tuning.

### 1.6 Novel contributions

We propose a new flexible HMM architecture that relaxes the modeling assumptions of existing HMM-based methods in two ways. First, we allow to choose, for each histone modification, a different discrete distribution type from a broad selection (Table 1). This allows us to model more flexible read count distribution shapes, including overdispersed and skewed distributions. Second, we provide flexible duration modeling (using an automated state extension technique) to capture the characteristics of broad and narrow chromatin domains in a single segmentation with nucleosome resolution. By applying our method to publicly available ChIP-seq data, we show that such a flexible HMM leads to a better model fit and may increase segmentation accuracy and biological interpretability.

**Table 1.** Overview of available discrete distributions (Johnson et al. 1993).

Name	Parameters
Binomial	n∈N,p∈[0,1]
Poisson	$\lambda \in R^{+}$
Negative binomial	$r\in R^{+},p\in [0,1]$
Beta binomial	$n\in N,\alpha \in R^{+},\beta \in R^{+}$
Beta negative binomial	$r\in R^{+},\alpha \in R^{+},\beta \in R^{+}$
Sichel	$\mu \in R^{+},\sigma \in R^{+},v\in R$

## 2 Methods

### 2.1 Probabilistic model

An HMM is formally defined as a quintuple (N,π,Σ,A,B), where {1,2,…,N} is a finite set of hidden states, *π* is a probability vector with the starting probabilities for each state, Σ is a set of observable emission values, *A* is an *N *×* N* matrix, where each entry *a_ij_* denotes the transition probability to move from state *i* to state *j*, and $B=\left(b_{j}(\cdot )\;|\;j\in \{1,2,\ldots ,N\}\right)$ is the complete collection of parameters required to calculate the emission probabilities of an observation in each state *j* (Lee and Park 2014). For a sample with *T* observations or time points, the collection of random variables is thus given by (*O*, *Q*), where $O=(O_{1},\ldots ,O_{T})$ denotes the observed sequence and $Q=(Q_{1},\ldots ,Q_{T})$ denotes the hidden state sequence.

Due to the HMM independence assumptions [*Q* is a Markov chain, *O_t_* is conditionally independent of everything else, given *Q_t_*; Bilmes (1998)], the probability that an HMM with parameters θ=(π,A,B) generates the combination (Q=q,O=o) is

(1) $$P_{\theta }(Q=q,O=o)=\pi_{q_{0}}\prod_{t=1}^{T}a_{q_{t-1}q_{t}}b_{q_{t}}(o_{t}).$$

For given fixed *O *=* o*, the Viterbi algorithm (Rabiner 1989) determines the state sequence *q* that maximizes this probability (for fixed transition and emission probabilities).

For chromatin segmentation, the emission alphabet is given by the multivariate, countable infinite set

(2) $$\Sigma =\{(x_{1},\ldots ,x_{M})\;|\;x_{m}\in N_{0},m\in \{1,\ldots ,M\}\},$$

where *M* is the dimension of the emission alphabet, corresponding to the number of histone modifications in the input data. Hence, the natural choice is to model the emission probabilities using a multivariate discrete distribution. Under the assumption that the read counts of all histone modifications are conditionally independent of each other given a state, the emission probability for an observation $o_{t}=(o_{t1},\ldots ,o_{tM})$ in state *j* is given by

(3) $$b_{j}(o_{t})=P_{\theta }(O_{t}=o_{t}\;|\;Q_{t}=j)$$

(4) $$=P_{\theta }(O_{t1}=o_{t1},\ldots ,O_{tM}=o_{tM}\;|\;Q_{t}=j)$$

(5) $$=\prod_{m=1}^{M}P_{\theta }(O_{tm}=o_{tm}\;|\;Q_{t}=j).$$

This leads to a flexible framework in which different distribution types may be selected to model the read counts of distinct histone marks. Table 1 gives an overview over the univariate discrete distributions available in EpiSegMix (see Supplementary Section S1 for details). Distributions with more parameters are more flexible and hence lead to a more accurate model fit: One-parameter distributions (Poisson) may fit the mean of the observed read counts, but not variance or skewness; two-parameter distributions (e.g. Negative Binomial) may fit both mean and variance but not skewness; three-parameter distributions may fit all three moments. With data from several thousand genomic intervals per state, there is no danger of overfitting three parameters. Still, each distribution has its own limitations and dependencies between moments, so having a variety of options is beneficial. The best performing distribution may vary for histone modifications and between experiments with different data quality. Therefore, we provide a workflow to find for each mark the distribution that maximizes the log-likelihood of a three-state HMM with univariate emissions. For further information, see Supplementary Section S4.

### 2.2 Duration modeling

**Figure 2.**
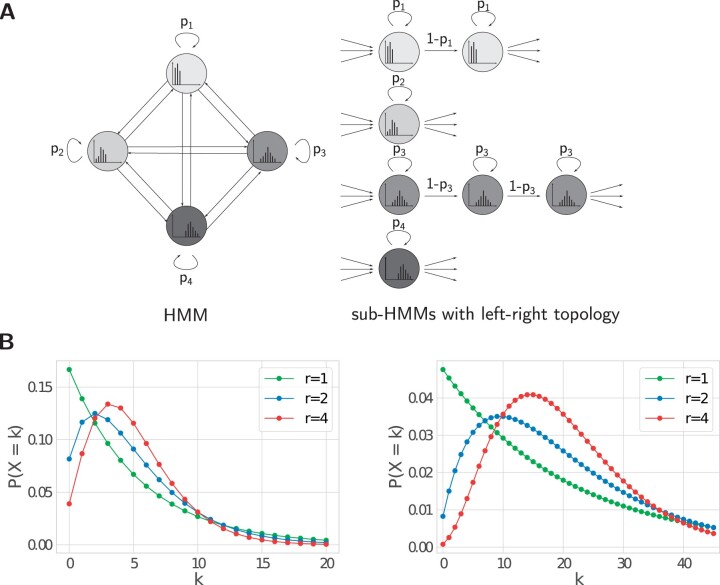
Duration modeling. (A) Extended-state HMM with different numbers of states in the sub-HMMs for a univariate four-state HMM. (B) Comparison of state duration distribution for a state in a classical HMM (*r *=* *1) and in a topology HMM with two or four sub-states to achieve the same mean of 5 (left) or 20 (right).

The typical HMM topology is a fully connected graph, including self-loops on states. Hence the sojourn time *X* in a state follows a Geometric distribution $P(X=k)=p\cdot {\left(1-p\right)}^{k-1}$ for some p>0, with exponentially decreasing probabilities for longer durations (Rabiner 1989). Although the mean of a Geometric distribution can be made arbitrarily large, the variance increases with it and the mode stays at P(X=1). Geometric distributions model durations that are short with higher probability, but have limited flexibility when modeling longer durations with a mode far away from 1. We therefore propose to use an extended-state HMM architecture, in which each state is internally represented as a sub-HMM. Each sub-HMM has a linear left-to-right topology with a different number of sub-states, but with the additional constraints that all sub-states have the same emission probabilities and same self-transition probabilities (Russell and Cook 1987) (see Fig. 2A). With this topology, the state duration is the sum of a number of independent Geometrically distributed random variables, which has a Negative Binomial distribution. It has two parameters: the state-exit probability *P* (same as for a Geometric distribution), and the copy number *r* (where *r *=* *1 gives the Geometric distribution). In contrast to a Geometric distribution, the mean and variance of a Negative Binomial distribution can be controlled independently, and the mode can be placed at an arbitrary duration (Fig. 2B). Therefore, both long and short durations can be modeled flexibly.

The number of sub-states is fitted automatically during parameter estimation. Starting from a standard ergodic topology (each state is represented by one sub-state), the segment lengths are computed for each state under the current model. The number of sub-states is then given by the estimated parameter *r* of a Negative Binomial distribution under the additional constraint that *r* is a natural number between 1 and 5. Each state may hence have a different number of sub-states depending on the observed segment lengths. By default, the topology is adjusted twice. Before the adjustments, the parameters of the HMM are estimated for a fixed number of iterations (default 5) and until convergence for the final topology.

### 2.3 Parameter estimation

Before an HMM can be used to estimate the state sequence, its parameters (transition and emission probabilities) must be estimated. As we typically do not have labeled training data, parameter estimation must proceed in an unsupervised manner using the Baum–Welch algorithm, which is a concretization of the general expectation maximization (EM) algorithm tailored to the structure of HMMs (Rabiner 1989). It alternates between estimating the model parameters (M-step), given a (fuzzy or probabilistic) assignment of observations to states, and re-estimating the state membership probabilities of each observation (E-step) until convergence (details in Supplementary Section S1).

## 3 Implementation

The flexible distribution HMM and parameter estimation is implemented in C++ as a command-line tool EpiSegMix. Its source code is at https://gitlab.com/rahmannlab/episegmix.

All steps of the surrounding workflow are incorporated in a Snakemake workflow (Mölder *et al.* 2021).

By default, we estimate parameters on the ENCODE pilot regions which contain a good representation of the whole genome and are thus commonly used to fit the model (Hoffman *et al.* 2012, Daneshpajouh *et al.* 2022). Alternatively, the user may specify a list of chromosomes to be used for model fitting. The segmentation is performed genome-wide.

The main output of chromatin segmentation is a file that assigns one state to each position in the genome, and an HTML report with plots that characterize the model and segmentation, enabling their biological interpretation. For example, the heatmap showing the normalized histone modification intensities of each state (as in Fig. 6B) is central to determine the genomic function of the states.

## 4 Results

We evaluated EpiSegMix on publicly available ChIP-seq data for the human cell lines K562, HepG2, GM12878, IMR90, H1, and SJCRH30 provided by the ENCODE consortium (Dunham *et al.* 2012) using the most recently processed data. With the selected cell lines, we evaluate our method on ChIP-seq experiments that were performed over the last decade (from 2010 to 2020) with different data properties (e.g. the mapped read length ranges from 36 to 100 bp). To analyze the robustness of all methods, we generated the count matrix for two replicate experiments each (see Supplementary Section S6). To reproduce the results, all accession numbers are provided in Supplementary Section S9 and the script to create the count matrix is part of the code repository. We restricted our analysis to the six core histone marks H3K9me3, H3K27me3, H3K36me3, H3K4me1, H3K4me3, and H3K27ac defined by the IHEC consortium (Bujold *et al.* 2016).

Since chromatin segmentation is an unsupervised method and no ground truth is available, we evaluate the performance of EpiSegMix by comparing it to three established chromatin segmentation tools ChromHMM (Ernst and Kellis 2012), EpiCSeg (Mammana and Chung 2015), and Segway (Chan *et al.* 2018).

We first perform a quantitative comparison by evaluating how well the different methods can predict gene expression and ATAC-seq data. Afterwards, we analyze how the different methods reflect known genome biology by showing further characteristic plots for one exemplary dataset.

### 4.1 Data processing

In a preprocessing step, we convert the aligned reads into a count matrix using the *bamsignals* package (Mammana and Helmuth 2023). In the count matrix, each row corresponds to a consecutive, nonoverlapping region with a fixed window size (default 200 base pairs), called bins, and each column corresponds to a distinct histone mark. Each read is assigned to exactly one genomic bin depending on the position of its 5’ end.

For all methods, we fitted a 10-state model. EpiSegMix and Segway were trained on the ENCODE pilot regions of *hg38*. For EpiSegMix, three-state HMMs were fitted individually for each mark and different distributions. We then chose the distribution with the highest log-likelihood, listed in Supplementary Section 4. For Segway, the *resolution* was set to 200 bp, the *track-weight* to 0.01, and the *segtransition-weight-scale* and *prior-strength* to 1. For ChromHMM and EpiCSeg, the default parameters were used (200 bp resolution, 10-state model).

### 4.2 Advantages of flexible distribution modeling

**Figure 3.**
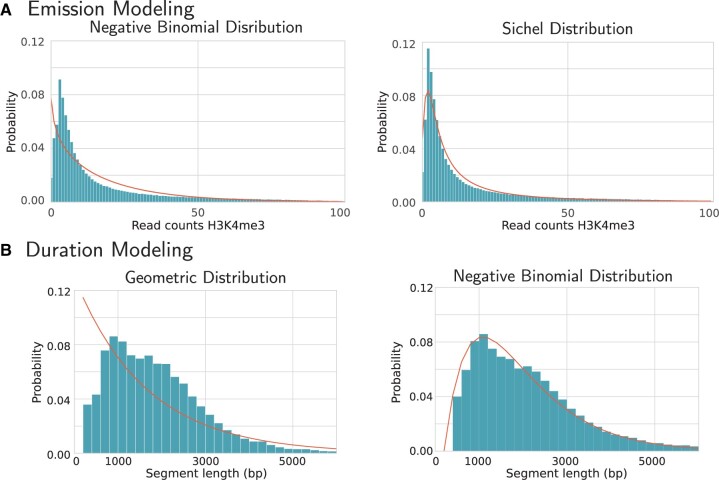
Flexible emission and duration modeling. The histograms show the sample distribution and the curves show the theoretical distribution fitted by the model. (A) Results of fitting a three-state HMM to the mark H3K4me3 using the Negative Binomial and Sichel distribution (for the state with high H3K4me3 in HepG2_1). (B) State duration in the HepG2_1 promoter state for an HMM with a classic (Geometric) and extended (Negative Binomial) topology.

To show the advantages of flexible emission and duration distribution types for chromatin segmentation, we compare fitted models using different emission distributions and with and without duration modeling. Narrow marks, such as H3K4me3, often have skewed read count distributions. Fitting the Negative Binomial (2 parameters) and Sichel (3 parameters) distribution to the read counts of H3K4me3 shows the limitation of the Negative Binomial distributions to model highly skewed and overdispersed data (Fig. 3A). Further evaluation of the effect that emission modeling has on the segmentation is provided in Supplementary Section S4.

Figure 3B shows that the state duration, determining the segment length (number of consecutive bins assigned to the same state), does not follow a Geometric distribution for most chromatin states. In comparison, the Negative Binomial distribution, as fitted by our flexible duration model, leads to a more accurate description of the real segment length distribution.

### 4.3 Evaluation of gene expression prediction

**Figure 4.**
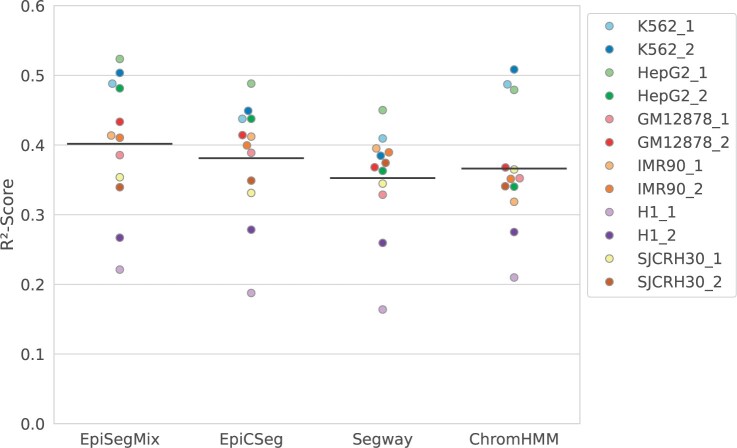
Prediction of transcription levels from chromatin state labels: coefficient of determination *R*^2^ (*y*-axis) using state labels as categorical predictors for log(FPKM+1) expression values for the different methods (*x*-axis) on different cell lines (color). Black lines indicate mean *R*^2^ for each method.

Since a biologically meaningful segmentation should have states that correlate with different gene expression levels, we compared how well the chromatin states of EpiSegMix, EpiCSeg, ChromHMM, and Segway can predict gene expression. To measure the gene expression in each 200 bp bin that contains (part of) a protein-coding gene, we used total RNA-seq experiments for the different cell lines provided by ENCODE and assigned each bin the log(FPKM + 1) normalized expression value of the gene (FPKM: fragments per kilobase of transcript per million mapped reads). We performed linear regression with the state labels as categorical predictors, i.e. for each bin *i’*s true expression *x_i_*, we used the state-specific mean expression as predictor $\hat{x_{i}}$ and measured the mean quadratic regression error versus the mean quadratic error using the global mean $\bar{x}$ as predictor and computed the coefficient of determination $R^{2}=1-[\sum_{i}{\left(x_{i}-\hat{x_{i}}\right)}^{2}]/[\sum_{i}{\left(x_{i}-\bar{x}\right)}^{2}]$ (between 0 and 1, where 1 is perfect). The *R*^2^ values vary for the different cell lines, which can partly be explained by the unequal data quality. Figure 4 compares the *R*^2^ values of the different methods across cell lines. On average, EpiSegMix has the highest predictive power among the methods.

### 4.4 Evaluation of ATAC-seq prediction

**Figure 5.**
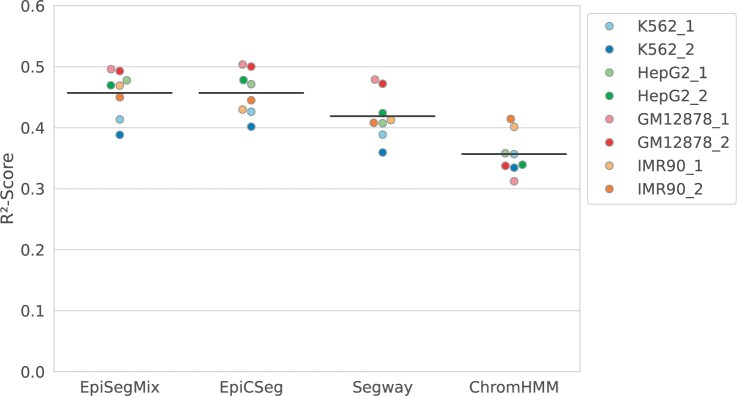
Prediction of ATAC-seq levels from chromatin state labels. The plot shows the coefficient of variation (*R*^2^) using standard linear regression with the state labels as categorical predictors to predict log  transformed ATAC counts. The black line shows the mean *R*^2^ value.

We performed a similar analysis predicting ATAC-seq read counts instead of gene expression levels for all cell lines with available ATAC-seq experiments. We counted how many reads of the ATAC-seq experiment map to each nonoverlapping 200 bp bin in the genome to generate a count matrix in the same way as for the histone counts. In the same way as for gene expression, we performed linear regression to predict log-transformed ATAC-seq read counts using the state labels as categorical predictors. Since ATAC-seq measures the chromatin accessibility, active states, like promoter or transcription states, should be predictive of high ATAC-seq counts, while heterochromatic states should be predictive of low ATAC-seq counts. Figure 5 shows that EpiSegMix and EpiCSeg have a similar predictive power of chromatin accessibility, as measured by the coefficient of determination *R*^2^, while ChromHMM and Segway have lower *R*^2^ scores on average.

### 4.5 Evaluation of reflected genome biology

**Figure 6.**
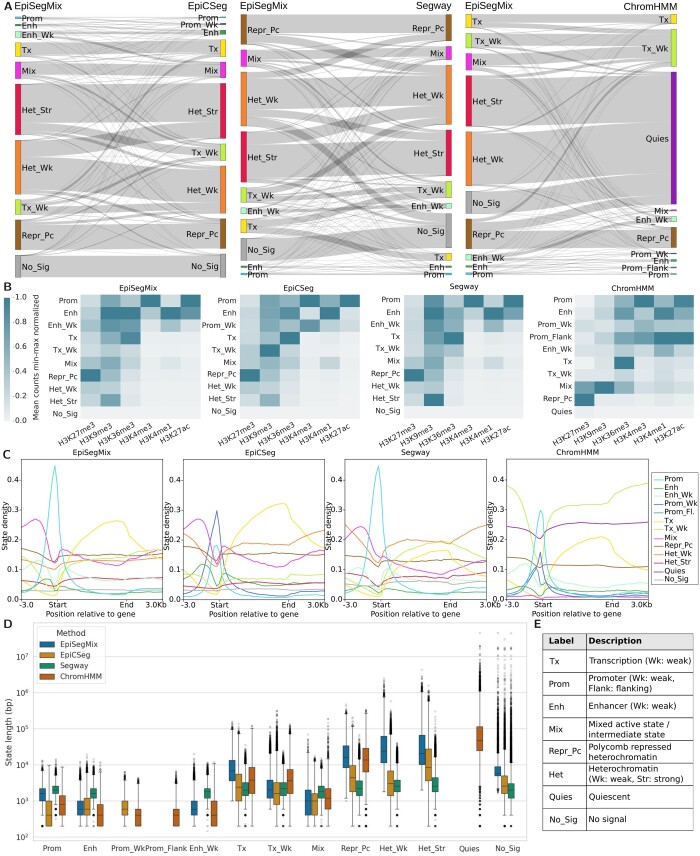
Comparison between EpiSegMix and EpiCSeg, Segway and ChromHMM for K562_1. (A) State overlap between methods. The bar heights correspond to the genomic coverage of the biologically annotated state and the edge thickness to the overlap between the methods. (B) Heatmaps showing the histone enrichment per state. The mean counts per state are normalized column wise, such that for each histone modification the state with the maximum mean count has a value of one and the state with the lowest mean count has a value of zero. (C) State distribution. The line plots show how often each state occurs around protein coding genes. (D) State duration. The plot shows for each biologically annotated state the state length distribution in base pairs of the different methods. (E) State description.

We perform a qualitative analysis of the different methods by comparing the similarities of the genome-wide segmentation and functional assignment of states, their genome coverage, overall segment length, and enrichment around protein coding genes (Fig. 6). An in-depth example (genome browser view) is provided in Supplementary Section S7. For better comparability, we manually assigned a label that best describes the biological function of the state to each numerical state ID. This facilitates the comparison of state assignments between methods. A description of each label is given in Fig. 6E and Supplementary Section S8.

Figure 6A shows that the states assigned by the count-based methods EpiSegMix, EpiCSeg, and Segway have a similar genome coverage and high overlap between state assignments. Moreover, all three methods EpiSegMix, EpiCSeg, and Segway provide a deeper epigenomic distinction of heterochromatic regions that ChromHMM aggregates into a single state. EpiSegMix discriminates a “no signal” state from a strong and a weak heterochromatic state that show distinctive patterns of enriched histone marks. Thereby, EpiSegMix better captures transitions from closed to open chromatin as compared to ChromHMM (Supplementary Section S7). This distinction is also supported by Segway and EpiCSeg. In addition, EpiSegMix defines a more consistent and accurate label for unmappable regions in comparison to all other methods (Supplementary Section S6). While, in a 10-state model, ChromHMM appears to provide a more fine-grained distinction of regulatory states, such as weak and flanking promoter regions, the robustness of these classifications is not always given. Thus, a biological interpretation of these regions should be taken with caution (Supplementary Section S6).

Figure 6B shows the genome wide enrichment of histone marks across states for each tool. All methods find similar patterns of histone marks: Enrichment of regulatory marks such as H3K27ac, H3K4me1, and H3K4me3, is predominantly observed in promoter and enhancer states, as expected. Actively transcribed mark H3K36me3 is enriched in transcription states; the repressive mark H3K27me3 is most strongly enriched in Polycomb repressed heterochromatin. Overall absolute levels of H3K9me3 are low, but due to the column-wise normalization in Fig. 6B, it appears enriched in a number of states, possibly reflecting overall background noise.

Despite the overall high state concordance across tools, some differences can be observed particularly in a gene centered comparison, i.e. across gene bodies, including ±3 kb upstream and downstream of the gene transcription start and gene end, respectively (Fig. 6C). Coordinates were taken from ENCODE reference ENCFF824ZKD. EpiSegMix and Segway show a higher enrichment of promoter states around TSSs as compared to ChromHMM and EpiCSeg, which discriminate between weak and strong promoters. This distinction is not linked to a better prediction of transcription levels.

When comparing the segment lengths (i.e. consecutive genomic bins assigned to the same state) and their genomic distributions across the different methods we observe that the duration flexibility of EpiSegMix helps to capture the wide range of short (promoters/enhancers), intermediate (short to long genes), and long (heterochromatic regions) state durations in a more consistent manner as compared to all other methods. For example, EpiSegMix’ Tx state most effectively covers large genes (i.e. over 40% of all human genes are longer than 0.8 × 10^4^ bp), allowing for a more accurate annotation of genes among different classes. Another advantage of EpiSegMix in comparison to EpiCSeg and Segway can be observed in the longer segment lengths for Polycomb repressed genes (Repr_Pc) and for heterochromatic regions (Het_Wk and Het_Str), which more closely match the known size of broad domains (Steensel and Belmont 2017).

## 5 Discussion

We developed EpiSegMix, a flexible HMM framework for chromatin segmentation. We enhanced the flexibility of existing HMMs with respect to modeling both emission probabilities and state durations. To account for the overdispersed and skewed ChIP-seq read count distributions, the read counts of each histone modification can follow a different discrete distribution type. We currently support a variety of distributions, and further distributions may be added in the future. The internal HMM topology was adjusted to be able to model state durations that follow a Negative Binomial instead of a Geometric distribution, which better reflects the inherent segment length of chromatin states that cover either small peaks or broad domains. For example, lamina-associated domains (LADs; heterochromatin at the nuclear periphery) usually have a size between 10^4^ and 10^7^ bp (Steensel and Belmont 2017).

A comparison with ChromHMM, Segway, and EpiCSeg suggests that EpiSegMix has the potential to provide segmentations that better reflect genomic annotations and yields states that are more predictive of gene expression. Moreover, the flexible duration modeling allows to effectively capture the reflective state lengths of long gene bodies and heterochromatic domains. The influence of the modified HMM topology on the segmentation suggests that testing other topology models is an important aspect to increase the modeling accuracy. Another direction of future work could be to combine the flexible duration modeling of EpiSegMix with a hierarchical HMM, as proposed by Marco *et al.* (2017), which may prove to be a powerful idea to perform inter-dependent chromatin segmentation at different length scales.

Although our results suggest that flexible distributions such as the Sichel or Beta Negative Binomial distribution often give the best results, we support a variety of distributions to deal with changing data properties and provide an easily extendable framework to integrate other data, such as ATAC-seq, DNase-seq or transcription factor ChIP-seq data.

In summary, we show that the modeling assumptions of the HMM have an impact on the segmentation quality and biological interpretation. Due to its high flexibility, EpiSegMix accurately fits HMM read count data with varying distributional properties and provides the additional option of flexible duration modeling. Finally, EpiSegMix provides a widely configurable framework for chromatin segmentation that can be applied to a wide range of data.

## Supplementary Material

btae178_Supplementary_Data

## Data Availability

The data can be downloaded from the ENCODE portal. All accession numbers are provided in Supplementary Section S9.

## References

[btae178-B1] Allis CD , Jenuwein T. The molecular hallmarks of epigenetic control. Nat Rev Genet 2016;17:487–500. 27346641 10.1038/nrg.2016.59

[btae178-B2] Baker M. Making sense of chromatin states. Nat Methods 2011;8:717–22. 21878916 10.1038/nmeth.1673

[btae178-B3] Bannister AJ , Kouzarides T. Regulation of chromatin by histone modifications. Cell Res 2011;21:381–95. 21321607 10.1038/cr.2011.22 PMC3193420

[btae178-B4] Barski A , Cuddapah S, Cui K et al High-resolution profiling of histone methylations in the human genome. Cell 2007;129:823–37. 17512414 10.1016/j.cell.2007.05.009

[btae178-B5] Beacon TH , Delcuve GP, López C et al The dynamic broad epigenetic (H3K4me3, H3K27ac) domain as a mark of essential genes. Clin Epigenet 2021;13:138. 10.1186/s13148-021-01126-1 PMC8264473 34238359

[btae178-B6] Bilmes J. A gentle tutorial of the EM algorithm and its application to parameter estimation for Gaussian mixture and hidden Markov models. ICSI Tech Rep Ser vol. TR-97-021, Berkeley, 1998.

[btae178-B7] Blackledge NP , Klose RJ. The molecular principles of gene regulation by polycomb repressive complexes. Nat Rev Mol Cell Biol 2021;22:815–33. 34400841 10.1038/s41580-021-00398-y PMC7612013

[btae178-B8] Bujold D , Morais DAdL, Gauthier C et al The international human epigenome consortium data portal. Cell Syst 2016;3:496–9.e2. 27863956 10.1016/j.cels.2016.10.019

[btae178-B9] Carter B , Zhao K. The epigenetic basis of cellular heterogeneity. Nat Rev Genet 2021;22:235–50. 33244170 10.1038/s41576-020-00300-0 PMC10880028

[btae178-B10] Cavalli G , Heard E. Advances in epigenetics link genetics to the environment and disease. Nature 2019;571:489–99. 31341302 10.1038/s41586-019-1411-0

[btae178-B11] Chan RCW , Libbrecht MW, Roberts EG et al Segway 2.0: gaussian mixture models and minibatch training. Bioinformatics 2018;34:669–71. 29028889 10.1093/bioinformatics/btx603 PMC5860603

[btae178-B12] Daneshpajouh H , Chen B, Shokraneh N et al Continuous chromatin state feature annotation of the human epigenome. Bioinformatics 2022;38:3029–36. 35451453 10.1093/bioinformatics/btac283 PMC9154241

[btae178-B13] Day N , Hemmaplardh A, Thurman RE et al Unsupervised segmentation of continuous genomic data. Bioinformatics 2007;23:1424–6. 17384021 10.1093/bioinformatics/btm096

[btae178-B14] Diaz A , Park K, Lim DA et al Normalization, bias correction, and peak calling for ChIP-seq. Stat Appl Genet Mol Biol 2012;11:Article 9. 10.1515/1544-6115.1750 PMC3342857 22499706

[btae178-B15] Dunham I , Kundaje A, Aldred SF et al An integrated encyclopedia of DNA elements in the human genome. Nature 2012;489:57–74. 22955616 10.1038/nature11247 PMC3439153

[btae178-B16] Ernst J , Kellis M. Discovery and characterization of chromatin states for systematic annotation of the human genome. Nat Biotechnol 2010;28:817–25. 20657582 10.1038/nbt.1662 PMC2919626

[btae178-B17] Ernst J , Kellis M. ChromHMM: automating chromatin-state discovery and characterization. Nat Methods 2012;9:215–6. 22373907 10.1038/nmeth.1906 PMC3577932

[btae178-B18] Hoffman MM , Buske OJ, Wang J et al Unsupervised pattern discovery in human chromatin structure through genomic segmentation. Nat Methods 2012;9:473–6. 22426492 10.1038/nmeth.1937 PMC3340533

[btae178-B19] Johnson NL , Kotz S, Kemp AW et al Univariate discrete distributions, 2nd edn. New York, NY: Wiley, 1993.

[btae178-B20] Lee K-E , Park H-S. A review of three different studies on hidden Markov models for epigenetic problems: a computational perspective. Genomics Inform 2014;12:145–50. 25705151 10.5808/GI.2014.12.4.145 PMC4330247

[btae178-B21] Libbrecht MW , Chan RCW, Hoffman MM. Segmentation and genome annotation algorithms for identifying chromatin state and other genomic patterns. PLoS Comput Biol 2021;17:e1009423. 34648491 10.1371/journal.pcbi.1009423 PMC8516206

[btae178-B22] Mammana A , Chung H-R. Chromatin segmentation based on a probabilistic model for read counts explains a large portion of the epigenome. Genome Biol 2015;16:151. 26206277 10.1186/s13059-015-0708-z PMC4514447

[btae178-B23] Mammana A , Helmuth J. *bamsignals: Extract Read Count Signals from Bam Files*. R package version 1.34.0, 2023. https://bioconductor.org/packages/bamsignals.

[btae178-B24] Marco E , Meuleman W, Huang J et al Multi-scale chromatin state annotation using a hierarchical hidden Markov model. Nat Commun 2017;8:15011. 28387224 10.1038/ncomms15011 PMC5385569

[btae178-B25] Mölder F , Jablonski KP, Letcher B et al Sustainable data analysis with snakemake. F1000Res 2021;10:33. 34035898 10.12688/f1000research.29032.1 PMC8114187

[btae178-B26] Park PJ. ChIP–seq: advantages and challenges of a maturing technology. Nat Rev Genet 2009;10:669–80. 19736561 10.1038/nrg2641 PMC3191340

[btae178-B27] Rabiner L. A tutorial on hidden Markov models and selected applications in speech recognition. Proc IEEE 1989;77:257–86.

[btae178-B28] Russell M , Cook A. Experimental evaluation of duration modelling techniques for automatic speech recognition. In: *ICASSP ’87. IEEE International Conference on Acoustics, Speech, and Signal Processing, Dallas, TX, USA*, Vol. 12, New York, NY, USA: IEEE (Institute of Electrical and Electronics Engineers) 1987, 2376–9.

[btae178-B29] Steensel B. V , Belmont AS. Lamina-Associated domains: links with chromosome architecture, heterochromatin, and gene repression. Cell 2017;169:780–91. 28525751 10.1016/j.cell.2017.04.022 PMC5532494

