# Supplementary material for: EpiSegMix: a flexible distribution hidden Markov model with duration modeling for chromatin state discovery
Source: Bioinformatics. 2024 Apr 2;40(4):btae178. doi: 10.1093/bioinformatics/btae178 (PMC11026141; doi:10.1093/bioinformatics/btae178)
Supplement: btae178_Supplementary_Data [file btae178_supplementary_data.pdf]

Supplement

EpiSegMix: A Flexible Distribution Hidden Markov Model with Duration Modeling for Chromatin State Discovery

Johanna Elena Schmitz, Nihit Aggarwal, Lukas Laufer, Jörn Walter, Abdulrahman Salhab, and Sven Rahmann

January 2024

S1 Supplementary Methods

S1.1 Notation for Hidden Markov Models (HMMs)

- finite set of hidden states $\{1, 2, \ldots, N\}$
- emission alphabet Σ
- observation $O = (O_1, \ldots, O_T)$ with T time points, where T is the number of rows in the count matrix
- hidden state sequence $Q = (Q_1, \ldots, Q_T)$
- probability vector $(\pi_i)_{i \in \{1,2,\ldots,N\}}$, which defines the starting probabilities for all states
- $N \times N$ matrix A, where each entry a_{ij} denotes the transition probability to move from state i to state j
- state-specific emission parameters $B = \{b_j(\cdot) \mid j \in \{1, 2, \ldots, N\}\}$
- probability distribution $P_\theta(\cdot)$, governed by a set of parameters θ

S1.2 Parameter Estimation (Baum-Welch Algorithm)

The parameter estimation for an HMM with parameters $\theta = (\pi, A, B)$ is performed using the Baum-Welch algorithm (Rabiner, 1989), which is an expectation-maximization algorithm tailored to HMMs. In each iteration r, the complete log likelihood function is maximized until convergence to a local optimum or until the maximum number of iterations has been reached. In our flexible distribution HMM, the complete log-likelihood function is given by Equation (1)

$$\theta^{(r+1)} = \arg\max_\theta \left(\sum_{i=1}^{N} \gamma_i(1) \log \pi_i + \sum_{t=2}^{T} \sum_{i=1}^{N} \sum_{j=1}^{N} \xi_{ij}(t) \log a_{ij} + \sum_{t=1}^{T} \sum_{i=1}^{N} \gamma_i(t) \log b_i^{(\eta_i)}(o_t) \right), \quad (1)$$

where the emission probabilities $b_i^{(\eta_i)}(\cdot)$ are specified by the distribution type and its state-specific parameters η_i and

$$\gamma_i(t) = P_\theta(Q_t = i \mid O), \qquad i \in \{1, \ldots, N\}, t \in \{1, \ldots, T\}, \quad (2)$$

$$\xi_{ij}(t) = P_\theta(Q_t = i, Q_{t+1} = j \mid O), \qquad i, j \in \{1, \ldots, N\}, t \in \{1, \ldots, T\}, \quad (3)$$

which are efficiently computed using the forward-backward algorithm (Rabiner, 1989), resulting in the following update rules (Bilmes, 1998):

$$\pi_i^{(r+1)} = \gamma_i(1) \tag{4}$$

$$a_{ij}^{(r+1)} = \frac{\sum_{t=1}^{T-1} \xi_{ij}(t)}{\sum_{t=1}^{T-1} \gamma_i(t)} \tag{5}$$

$$\eta_i^{(r+1)} = \arg\max_{\eta_i} \sum_{t=1}^{T} \gamma_i(t) \log b_i^{(\eta_i)}(o_t) \tag{6}$$

Multivariate observations (histone marks) For an observation with M histone marks, the update formula for the emission parameters in state i is given by

$$\arg\max_{\eta_i} \sum_{t=1}^{T} \gamma_i(t) \log b_i^{(\eta_i)}(o_t) = \arg\max_{\eta_i} \sum_{t=1}^{T} \gamma_i(t) \log \left(\prod_{m=1}^{M} b_{im}^{(\eta_{im})}(o_t) \right) \tag{7}$$

$$= \arg\max_{\eta_i} \sum_{m=1}^{M} \sum_{t=1}^{T} \gamma_i(t) \log b_{im}^{(\eta_{im})}(o_t) \tag{8}$$

Thus, the parameters η can be optimized independently for each state i and each histone mark m:

$$\eta_{im}^{(r+1)} = \arg\max_{\eta_{im}} \sum_{t=1}^{T} \gamma_i(t) \log b_{im}^{(\eta_{im})}(o_t) \tag{9}$$

Numerical Maximum-likelihood optimization To guarantee the convergence of the Baum-Welch algorithm to a local optimum, the maximum likelihood estimators are used to update the parameters of all distributions. Since for most distributions no closed-form solution exists for Equation (9), it is optimized numerically using the *MIGRAD()* function from the *ROOT Minuit 2* package (Brun and Rademakers, 1997). All computations are performed with logarithms of probabilities for numerical stability.

Extended-state HMM In the extended-state HMM, for each state i, we create a sub-HMM with states $i_1, i_2, \ldots, i_{S_i}$, where S_i is the number of states in the sub-HMM of state i. The update formulas enforcing the additional constraints of equal emission and self-transition probabilities are given by

$$\theta_{im}^{(r+1)} = \arg\max_{\theta_{im}} \sum_{t=1}^{T} \left(\sum_{s=1}^{S_i} \gamma_{i_s}(t) \right) \log b_{im}^{(\eta_{im})}(o_t) \tag{10}$$

$$a_{ii}^{(r+1)} = \frac{\sum_{t=1}^{T-1} \sum_{s=1}^{S_i} \xi_{i_s i_s}(t)}{\sum_{t=1}^{T-1} \sum_{s=1}^{S_i} \gamma_{i_s}(t)} \tag{11}$$

All other parts of the Baum–Welch algorithm remain unchanged.

Initialization As an iterative technique, the Baum-Welch algorithm is sensitive to the given initial parameters. Therefore, systematically guessed initial values are beneficial both for convergence speed and goodness-of-fit. To find proper starting values, we first perform k-means clustering with k equal to the desired number of states. In the next step, the method-of-moments estimators are calculated for each cluster and used to initialize the emission parameters. The transition probabilities are initialized uniformly.

S1.3 Details on Available Emission Distributions

This section lists technical details about the available distributions for modeling the read counts (emissions of the HMM). We provide the probability mass function, the method-of-moments estimators for initialization and, if available, the closed form of maximum likelihood estimators (MLEs) for all implemented discrete distributions.

For the method-of-moments estimators, the sample mean $\bar{x}$ and sample variance s^2 for a sample $(x_1, \ldots, x_N)$ are given by

$$\bar{x} = \frac{1}{N} \sum_{i=1}^{N} x_i \tag{12}$$

$$s^2 = \frac{1}{N-1} \sum_{i=1}^{N} (x_i - \bar{x})^2 \tag{13}$$

S1.3.1 Binomial Distribution

The probability mass function of a Binomial distribution (BI) with parameters $0 \leq p \leq 1$ and a natural number $n > 0$ is given by (Bernoulli, 1713)

$$P(X = k) = \binom{n}{k} p^k (1-p)^{n-k} \quad \text{for } k = 0, 1, 2, \ldots, n\,, \tag{14}$$

where $\binom{n}{k}$ is the Binomial coefficient defined as $\frac{n!}{k!(n-k)!}$. Assuming that n is known, the method-of-moments estimator of p is given by

$$\hat{p} = \frac{\bar{x}}{n} \tag{15}$$

The MLE of p in state i is given for an observation $O = (o_1, \ldots, o_t)$ and membership coefficients $\gamma_i(t)$ by

$$\hat{p}_i = \frac{\sum_{t=1}^{T} \gamma_i(t) \cdot o_t}{n \cdot \sum_{t=1}^{T} \gamma_i(t)} \tag{16}$$

If n and p are unknown, the estimator of n is not guaranteed to be finite. Thus, we use the sample maximum as an estimator of n, as proposed by Fisher (1941).

S1.3.2 Poisson Distribution

The probability mass function of a Poisson distribution (PO) with parameter $\lambda > 0$ is given by (Poisson, 1837)

$$P(X = k) = \frac{\lambda^k \cdot e^{-\lambda}}{k!} \text{ for } k = 0, 1, 2, \ldots\,. \tag{17}$$

The method-of-moments estimator for λ is given by the sample mean (Johnson et al., 1993, ch. 4), and the MLE is computed for an observation $O = (o_1, \ldots, o_t)$ and membership coefficients $\gamma_i(t)$ by

$$\hat{\lambda}_i = \frac{\sum_{t=1}^{T} \gamma_i(t) \cdot o_t}{\sum_{t=1}^{T} \gamma_i(t)} \tag{18}$$

S1.3.3 Negative Binomial Distribution

The probability mass function of a Negative Binomial distribution (NBI) with parameters $r \geq 0$ and $0 \leq p \leq 1$ is given by

$$P(X = k) = \binom{k + r - 1}{r - 1} (1-p)^k p^r \quad \text{for } k = 0, 1, 2, \ldots\,. \tag{19}$$

The method-of-moments estimators are given by (Lindén and Mäntyniemi, 2011)

$$\hat{r} = \frac{\bar{x}^2}{s^2 - \bar{x}}\,, \tag{20}$$

$$\hat{p} = \frac{\bar{x}}{s^2}\,. \tag{21}$$

S1.3.4 Beta Binomial Distribution

The probability mass function of a Beta Binomial distribution (BB) with parameters $\alpha > 0$, $\beta > 0$ and a natural number $n > 0$ is given by (Griffiths, 1973)

$$P(X = k) = \binom{n}{k} \frac{B(\alpha + k, n + \beta - k)}{B(\alpha, \beta)} \qquad \text{for } k = 0, 1, \ldots, n\,, \tag{22}$$

where

$$B(\alpha, \beta) = \frac{\Gamma(\alpha) \cdot \Gamma(\beta)}{\Gamma(\alpha + \beta)} \tag{23}$$

is the Beta function and

$$\Gamma(x) = \int_0^\infty t^{x-1} \mathrm{e}^{-t}\, \mathrm{d}t \qquad \text{for } x > 0 \tag{24}$$

is the the Gamma function (Johnson et al., 1993, p.4). Other names for the Beta Binomial distribution are Pólya or Negative Hypergeometric distribution. The method-of-moments estimators of α and β are given by

$$\alpha = \frac{\pi}{\theta}\,, \tag{25}$$

$$\beta = \frac{1 - \pi}{\theta}\,, \tag{26}$$

where π and θ are estimated by

$$\hat{\pi} = \frac{\bar{x}}{n}\,, \tag{27}$$

$$\hat{\theta} = \frac{\hat{s}^2 - n\hat{\pi}(1 - \hat{\pi})}{n^2\hat{\pi}(1 - \hat{\pi}) - \hat{s}^2}\,. \tag{28}$$

Similar to the Binomial distribution, if n is unknown, it is estimated by the sample maximum.

S1.3.5 Beta Negative Binomial Distribution

The probability mass function of a Beta Negative Binomial distribution (BNB) with parameters $r > 0$, $\alpha > 0$, $\beta > 0$ is given by (Johnson et al., 1993, ch. 6.2.3)

$$P(X = k) = \frac{\Gamma(k + r)}{\Gamma(k + 1)\Gamma(r)} \frac{B(\alpha + r, \beta + k)}{B(\alpha, \beta)} \qquad \text{for } k = 0, 1, \ldots\,, \tag{29}$$

where $B(\cdot, \cdot)$ denotes the Beta function (23) and $\Gamma(\cdot)$ denotes the Gamma function (24). Other names for the Beta Negative Binomial distribution are generalized Waring distribution, inverse Markov–Pólya distribution or Beta–Pascal distribution.

Since the n–th theoretical moment is only finite if $\alpha > n$ and in addition r and β are interchangeable (Rodríguez-Avi et al., 2007), a simplified approach is used to calculate the method-of-moment estimators. First, r and p are estimated under the assumption of a Negative Binomial distribution. The method-of-moments estimators of α and β are then estimated for a Beta distribution with the

sample mean given by p and the sample variance given by an arbitrarily chosen value of 0.05. For $\bar{x} = p$ and $s^2 = 0.05$, α and β are then given by (Schröder and Rahmann, 2017)

$$\psi = \frac{\bar{x} \cdot (1 - \bar{x})}{s^2} - 1 \tag{30}$$

$$\alpha = \bar{x} \cdot \psi \tag{31}$$

$$\beta = (1 - \bar{x}) \cdot \psi \tag{32}$$

Since the method-of-moments estimators are only used for initialization of the Baum-Welch algorithm, inaccurate estimates are acceptable.

S1.3.6 Sichel Distribution

The probability mass function of a Sichel distribution with parameters $\mu > 0$, $\sigma > 0$ and $-\infty < v < \infty$ is given by (Sichel, 1992)

$$P(X = k) = \frac{\mu^k \, K_{k+v}(\alpha)}{k! \, (\alpha \cdot \sigma)^{k+v} K_v\left(\frac{1}{\sigma}\right)} \quad \text{for } k = 0, 1, 2 \ldots , \tag{33}$$

where $\alpha = \sqrt{\frac{1}{\sigma^2} + \frac{2\mu}{\sigma}}$ and $K_v(t)$ denotes the modified Bessel function of the second kind of order v and argument t given by

$$K_v(t) = \frac{1}{2} \int_0^\infty x^{v-1} \mathrm{e}^{-\frac{1}{2}t(x+x^{-1})} \, \mathrm{d}x \, . \tag{34}$$

To avoid numerical problems during the computation of the Bessel function for large orders, the probability mass function is calculated recurrently based on the following recurrence relation (Stein et al., 1987)

$$P(X = 0) = \left(\frac{w}{\alpha}\right)^v \frac{K_v(\alpha)}{K_v(w)} , \tag{35}$$

$$P(X = 1) = P(X = 0) \cdot \frac{\mu \cdot w}{\alpha} \frac{K_{v+1}(\alpha)}{K_v(\alpha)} , \tag{36}$$

$$P(X = k) = \frac{2\mu w}{\alpha^2} \cdot \frac{k + v - 1}{k} \cdot P(X = k - 1) + \frac{(\mu w/\alpha)^2}{k(k-1)} \cdot P(X = k - 2) \tag{37}$$

with $w = \sqrt{\mu^2 + \alpha^2} - \mu$.

For $v = -\frac{1}{2}$ the Sichel distribution is equal to the inverse Gaussian Poisson distribution (Stein et al., 1987). For initialization, the parameters of the Sichel distribution are estimated using the method-of-moments estimators of the inverse Gaussian Poisson distribution, given by

$$v = -\frac{1}{2} , \tag{38}$$

$$\mu = \bar{x} , \tag{39}$$

$$\sigma = \frac{\sqrt{\alpha^2 + \bar{x}^2} + \bar{x}}{\alpha^2} , \tag{40}$$

with $w = \frac{\bar{x}}{s^2/\bar{x} - 1}$ and $\alpha^2 = (w + \mu)^2 - \mu^2$.

S1.3.7 Zero-Adjusted Distributions

A zero-adjusted distribution is a discrete mixture distribution that has a fixed zero probability π. We currently support the zero-adjusted Poisson, Negative Binomial, Beta Negative Binomial and Sichel

distribution. For a discrete random variable X', the zero adjusted probability mass function with zero probability $0 \leq \pi \leq 1$ is given by (Rigby et al., 2019)

$$P(X = k) = \begin{cases} \pi & k = 0 \\ (1-\pi) \cdot \frac{P(X'=k)}{1-P(X'=0)} & k \neq 0 \end{cases} \tag{41}$$

The method-of-moments estimator of π is given by the zero frequency and the MLE of π is given by

$$\hat{\pi}_i = \frac{\sum_{t=1}^{T} \gamma_i(t) \cdot \mathbb{1}_{o_t=0}}{\sum_{t=1}^{T} \gamma_i(t)} \tag{42}$$

where $\gamma_i(t)$ denotes the membership coefficient of observation o_t in state i. The parameters of the distribution for X' can be optimized independently.

S2 Selecting the Number of States

Since the log-likelihood of an HMM increases monotonically as the number of states increases, the selected number of states should be a good compromise between model fit and interpretability. Figure 1 shows the log-likelihood (LL) curve for different numbers of states on one of the cell lines. While the LL increases steeply for small numbers of states, it flattens for larger numbers; so 10 states emerges as a good compromise between goodness-of-fit and biological interpretability.

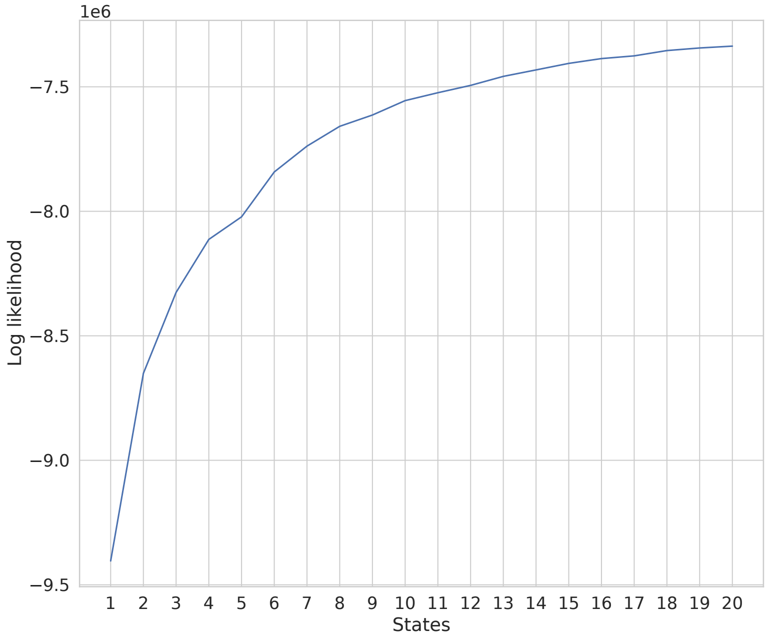

Figure 1: Log-likelihoods for a 1-state HMM up to a 20-state HMM for the IMR90_1 dataset using the six core histone marks H3K9me3, H3K27me3, H3K36me3, H3K4me1, H3K4me3 and H3K27ac. A higher log likelihood indicates a better model fit.

S3 Data Quality

Deeptools and *multiqc* were used to validate the quality of the data. All alignment files in BAM format were initially processed using *deeptools*. A multibam report was generated using *multiqc*. Figure 2 depicts the fingerprint curves of the genomic distribution of read counts of histone marks across various samples. A diagonal line would indicate a uniform enrichment of a histone mark across the genome (no selective enrichment). Broad marks such as H3K9me3 and H3K27me3 are expected to have curves

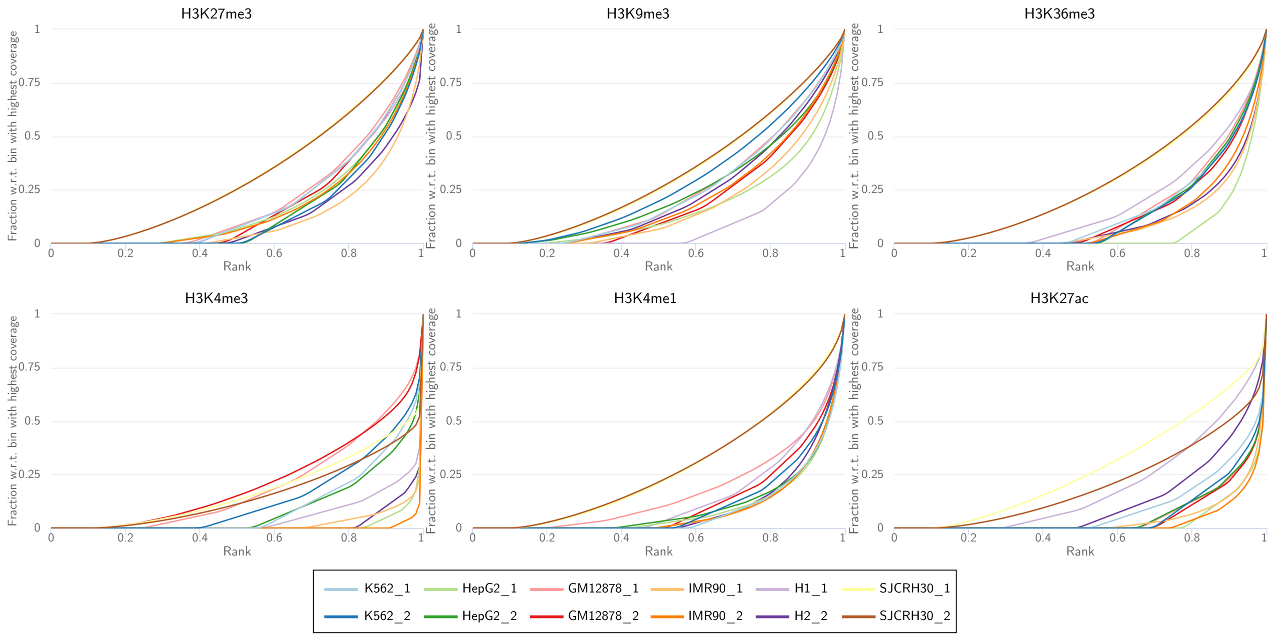

Figure 2: Quality of datasets by evaluating the genome-wide ChIP-seq enrichment for all histone marks (panels) in the different cell lines (colors). The curves show the fraction of reads (y-axis) needed to cover a certain fraction of the genome (x-axis). A diagonal line would indicate uniform enrichment of the histone mark across the genome (noise, input, no signal). A curve close to the x-axis and the vertical line at $x = 1$ indicates a highly selectively enriched histone mark. The fingerprint plots were created using *deeptools* (Fidel et al., 2016) and *multiqc* (Ewels et al., 2016).

near the diagonal line, since they are enriched in a large part of the genome. Conversely, narrow marks such as H3K27ac show a sharp curve called elbow point, where the peaks cover a small fraction of the genome. Figure 2 demonstrates that the data quality and amount of noise varies across the six cell lines and their replicates. Hence, our evaluation covers a broad spectrum of old and recent data of variable quality.

S4 Flexible Emission Modeling

S4.1 Selecting the Most Appropriate Distributions

To determine which distribution is best suited to model the read counts for each histone modification, we provide a workflow that tests, for each histone modification, a set of distributions and outputs a model file that can be used for the main segmentation workflow (`https://gitlab.com/rahmannlab/episegmix`). For each mark, a 3-state HMM is fitted individually for the different distributions. We then choose the distribution with the highest log-likelihood. Table 1 lists the used distribution for the results shown in the main article.

In most cases, the flexible three-parametric distributions (Sichel and Beta Negative Binomial distribution) were selected. In addition, the same distribution is not consistently chosen for the same histone mark which can be explained by the previously discussed differences in the data quality (see Supplementary Section S3).

Table 1: Selected distribution for each mark per dataset. NBI denotes the Negative Binomial, SI the Sichel and BNB the Beta Negative Binomial distribution. For cell lines SJCRH30_1 and GM12878_1, the Sichel distribution was used for all marks due to a better quality in the combined model.

	histone mark					
cell line	H3K9me3	H3K27me3	H3K36me3	H3K4me1	H3K4me3	H3K27ac
K561_1	SI	SI	SI	SI	SI	SI
K562_2	BNB	NBI	BNB	SI	BNB	SI
HepG2_1	BNB	BNB	BNB	SI	BNB	SI
HepG2_2	BNB	BNB	NBI	SI	SI	SI
H1_1	SI	BNB	BNB	SI	BNB	SI
H1_2	SI	BNB	SI	SI	SI	SI
SJCRH30_1	SI	SI	SI	SI	SI	SI
SJCRH30_2	SI	SI	SI	NBI	SI	BNB
IMR90_1	BNB	NBI	SI	SI	SI	SI
IMR90_2	BNB	NBI	BNB	SI	SI	SI
GM12878_1	SI	SI	SI	SI	SI	SI
GM12878_2	BNB	BNB	NBI	SI	BNB	SI

S4.2 Impact of Distribution Types

Figure 3 and Figure 4 show the advantage of using flexible emission modeling. Comparing the Log-Likelihood value between models with negative binomial and flexible emissions shows that the model fit of the flexible model is consistently better on all tested datasets. In addition, Figure 4 highlights that a theoretical better model fit also impacts the biological interpretability. In particular, narrow marks, such as H3K4me3, H3K4me1 and H3K27ac, can more accurately be modeled using flexible distributions, such as the Sichel distribution. For example, the Negative Binomial distribution cannot model the highly skewed read count distribution of H3K4me3 in state 3, where the high variance can only be modeled by selecting a parameter combination that additionally leads to a high probability for small read counts. In contrast, the Sichel distribution (as selected by the previously applied distribution fitting), is able to model the skewed and highly variable read counts of H3K4me3 without the above mentioned limitation. This also reflects in a lower genomic coverage of state 3. Since H3K4me3 is typically enriched in promoters while H3K4me1 is enriched in active enhancers, the results suggest that flexible distributions can better distinguish between promoters and enhancers.

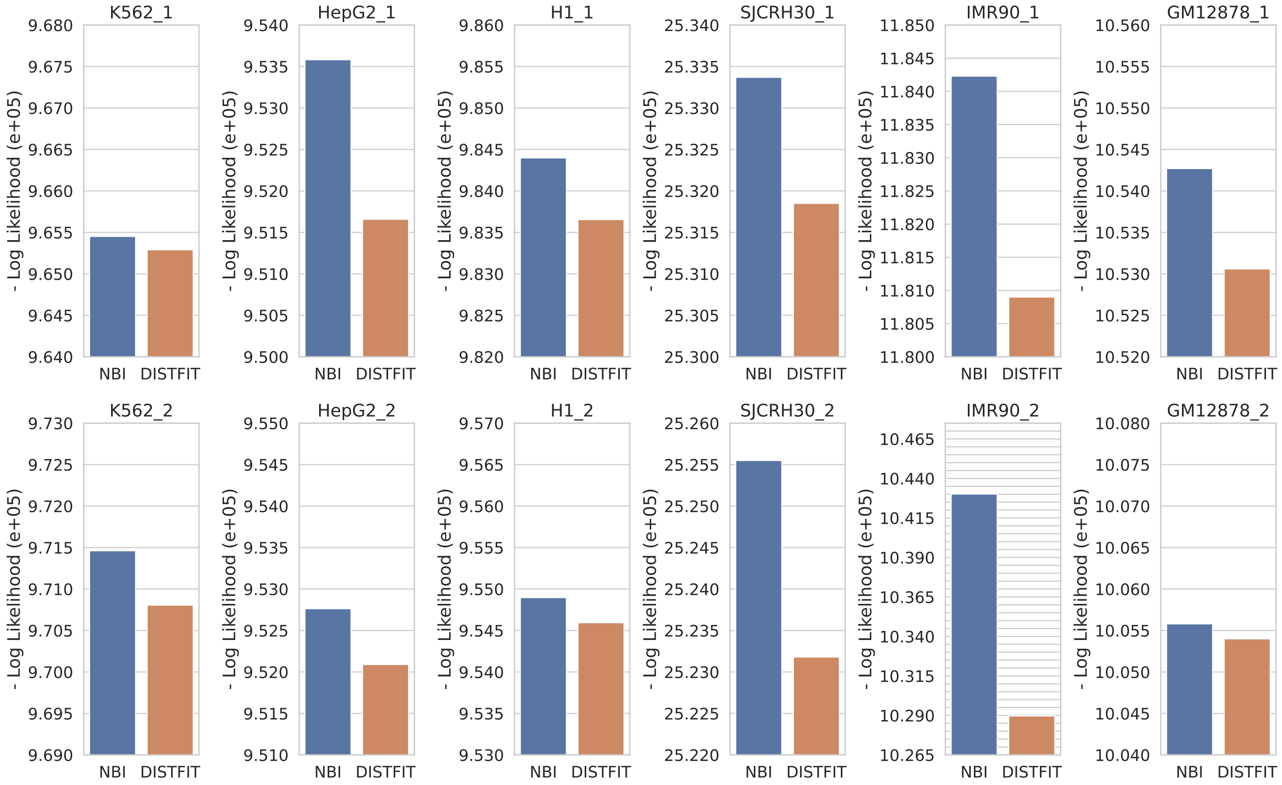

Figure 3: Comparison of the negative Log-Likelihood values between HMMs using Negative Binomial and flexible emissions. A lower negative Log-Likelihood value indicates a better model fit. For the *DISTFIT* model, the distributions were selected according to Table 1. Since the Log-Likelihood is only comparable across the same dataset, the plot is split by dataset. Note that the y-scale does not start at 0 to highlight the differences. Each grid line corresponds to an increase of $0.005 \cdot 10^5$.

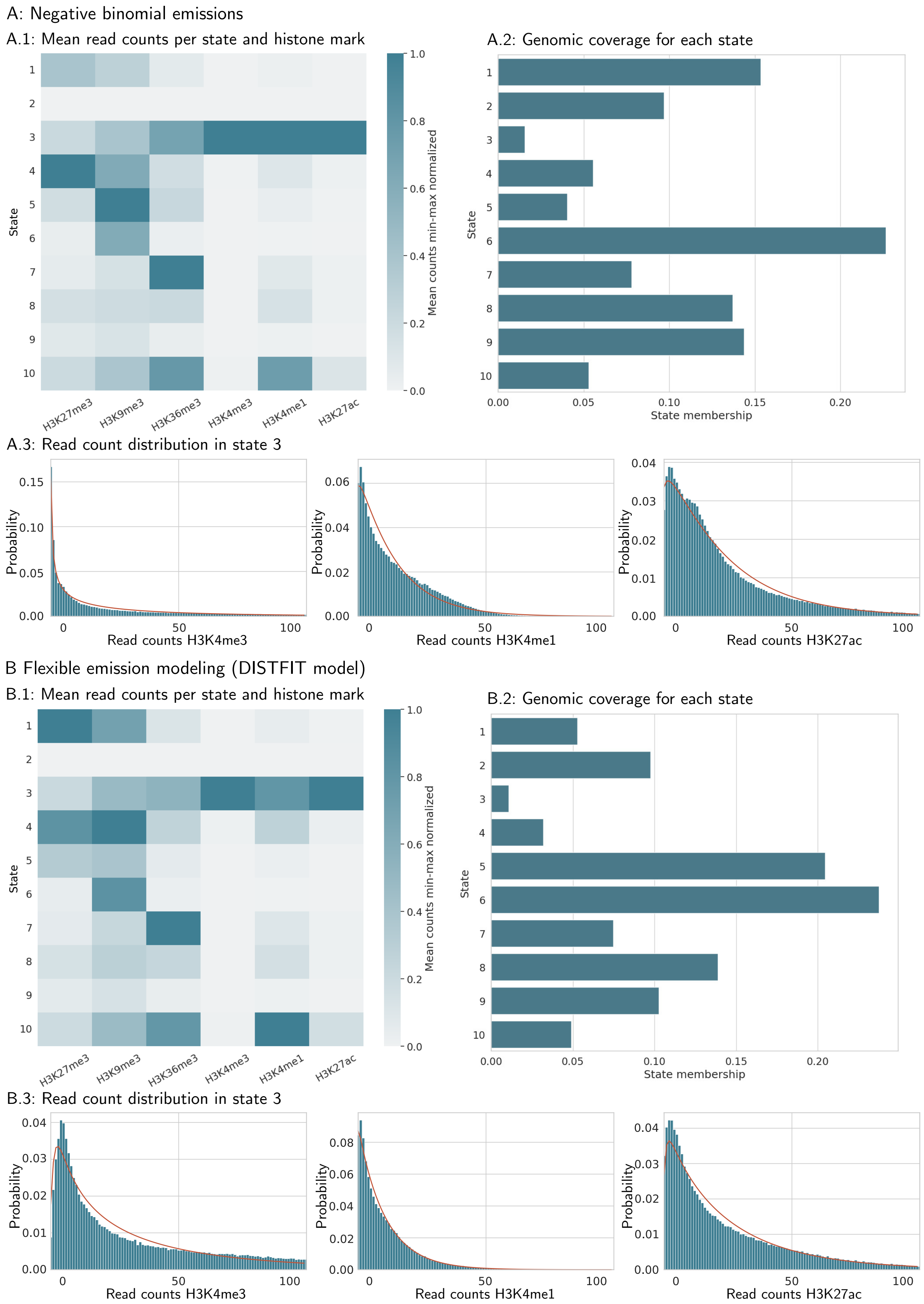

Figure 4: Effects of emission modeling on the discovery of chromatin states illustrated by IMR90_2 (**A**: negative binomial emissions vs. **B**: flexible emission modeling according to Table 1). In particular, the comparison of state 3 and 10 highlights the impact of choosing different distribution types.

S5 Impact of Duration Modeling

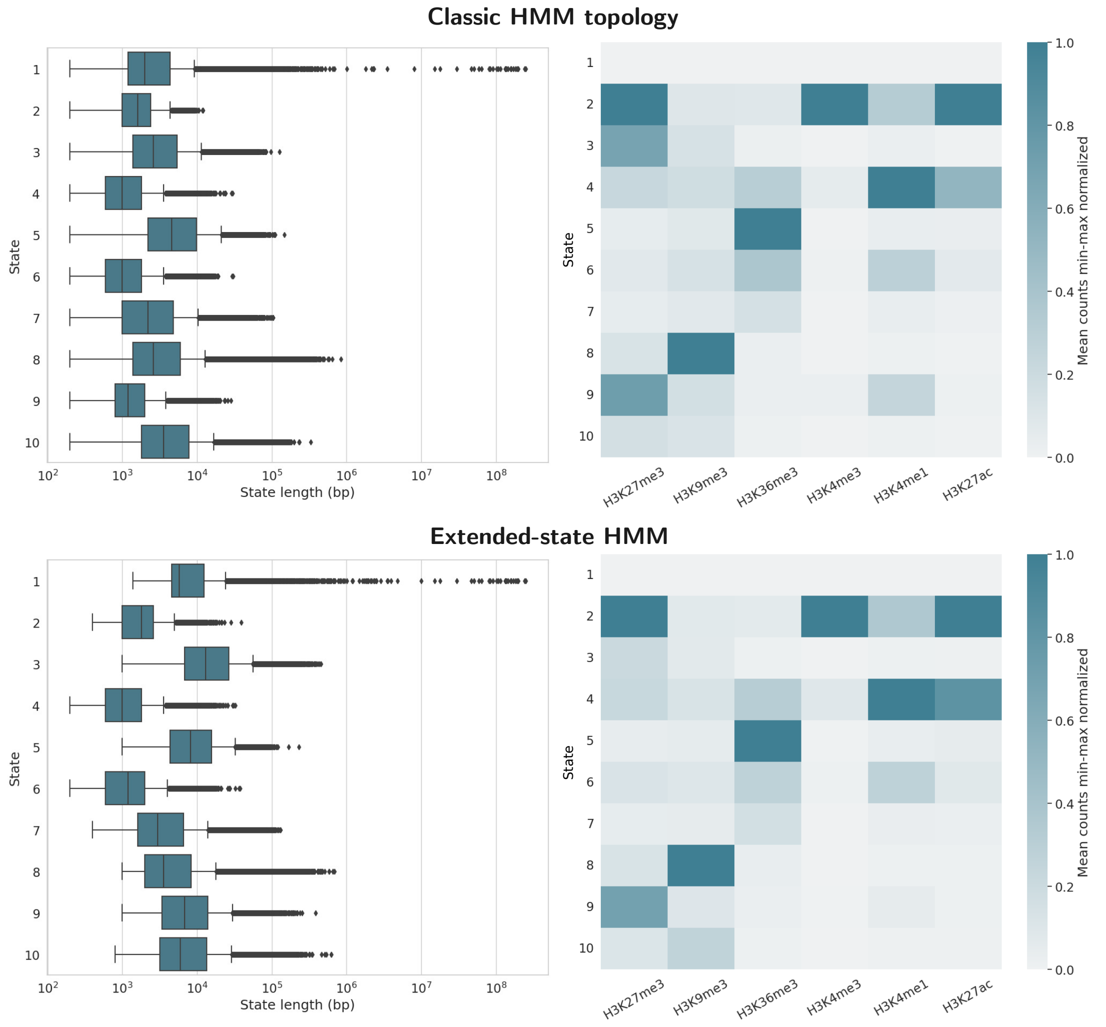

Figure 5: Comparison of segmentations trained with an HMM with classic topology and a flexible duration HMM (extended-state topology) for HepG2_1 using the emission distributions given in Table 1.

Figure 5 shows the segment length distribution (consecutive 200 bp bin assigned to the same state) and the normalized histone enrichment per state for an HMM with a classic topology (Geometric state duration) and an extended state HMM (Negative Binomial state duration). Both models show similar histone mark patterns but differing segment lengths. For example, the segment lengths of state 3 (heterochromatin, H3K27me3 and H3K9me3) and state 5 (transcriptional elongation, H3K36me3) are longer for the extended-state HMM in comparison to the classic HMM topology. Since the histone marks associated with active genes and heterochromatin are usually enriched as broad domains and not narrow peaks, we may presume that flexible duration modeling allows to more accurately model the length of biological domains.

S6 Robustness

To evaluate the robustness of the different chromatin segmentation methods, we generated the count matrix for two technical replicate experiments each. However, evaluating the robustness of chromatin segmentation methods depends on many factors that make general statements difficult. For example, a higher number of states usually results in a lower robustness due to interchangeable states. In addition, robustness often differs between active and repressive states (usually lower for regulatory states), which requires to evaluate the robustness after biological interpretation. Since so far no

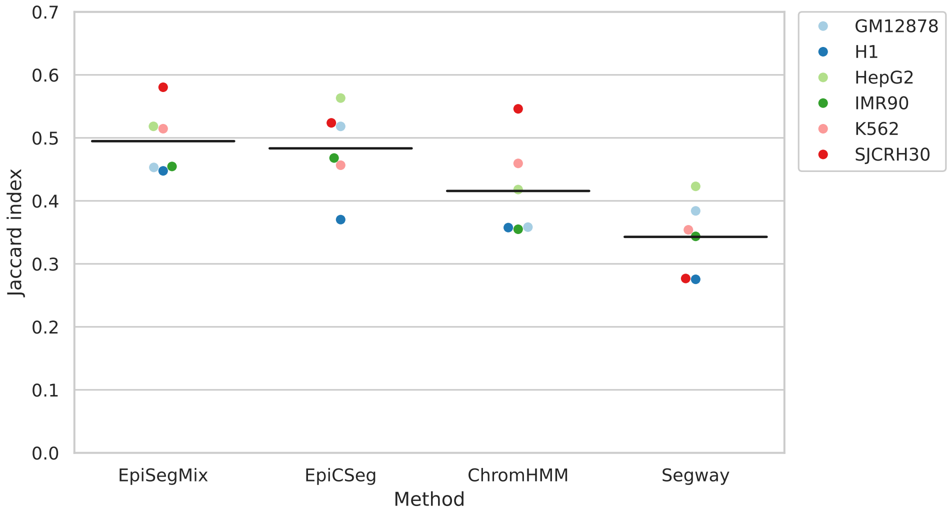

Figure 6: Robustness between replicate datasets. Each point represents the average Jaccard similarity between the segmentations of two technical replicates, with a higher Jaccard index indicating a greater overlap.

gold standard for automatic annotation exists, this approach may be flawed by manual or inaccurate automatic annotation. Hence, we compare the robustness using three different measures that capture different properties a robust method should fulfill.

First, we compared the Jaccard index, averaged over all states, between the segmentations of the two replicates after mapping the states of both segmentations according to the highest Jaccard index. Given a set of bins b_1 that are assigned to the state in the first replicate and a set of bins b_2 assigned to the corresponding state in the second replicate, the Jaccard index is defined as the size of the intersection $b_1 \cap b_2$ divided by the size of their union $b_1 \cup b_2$, and hence is between 0 (no agreement at all) and 1 (complete agreement). A higher index reflects higher robustness. Since this approach does not require a prior biological annotation, it is independent of potential biases during annotation. Figure 6 shows that the average robustness of EpiSegMix is slightly higher compared to the other methods which may be due to a high robustness in the heterochromatic states.

For a more detailed evaluation of the robustness we first manually annotated each state with one of 10 biological categories. Figure 7A shows the percentage of how many bases in the first replicate are covered by the closest matching state in the second replicate. Thus, if there existed a perfect one-to-one correspondence between both replicates each overlap would be 100%. In general, the robustness of all methods strongly varies between cell lines and biological annotations, which suggests that the robustness of all methods strongly depends on the data quality. EpiSegMix shows a higher robustness for promoter, transcription and strong heterochromatic states, but has a lower robustness for enhancers. However, due to the high variation we refrain from drawing a general conclusion.

Since a lower overlap may be caused by several states with very similar properties, we further calculated how many different states in the segmentation of the second replicate are required to cover over 95% of each state in the segmentation of the first replicate. Figure 7B shows the number of states for each annotated state, where a lower number of states indicates a higher reproducibility of finding states with a similar biological interpretation. In comparison to the other methods, EpiSegMix usually requires a similar or lower number of states to cover 95% of each state.

In conclusion, all methods have a limited robustness to replicate datasets, which may be caused by noise or a too fine-grained segmentation. In all considered measures, EpiSegMix had a similar (or sometimes better) robustness compared to other methods.

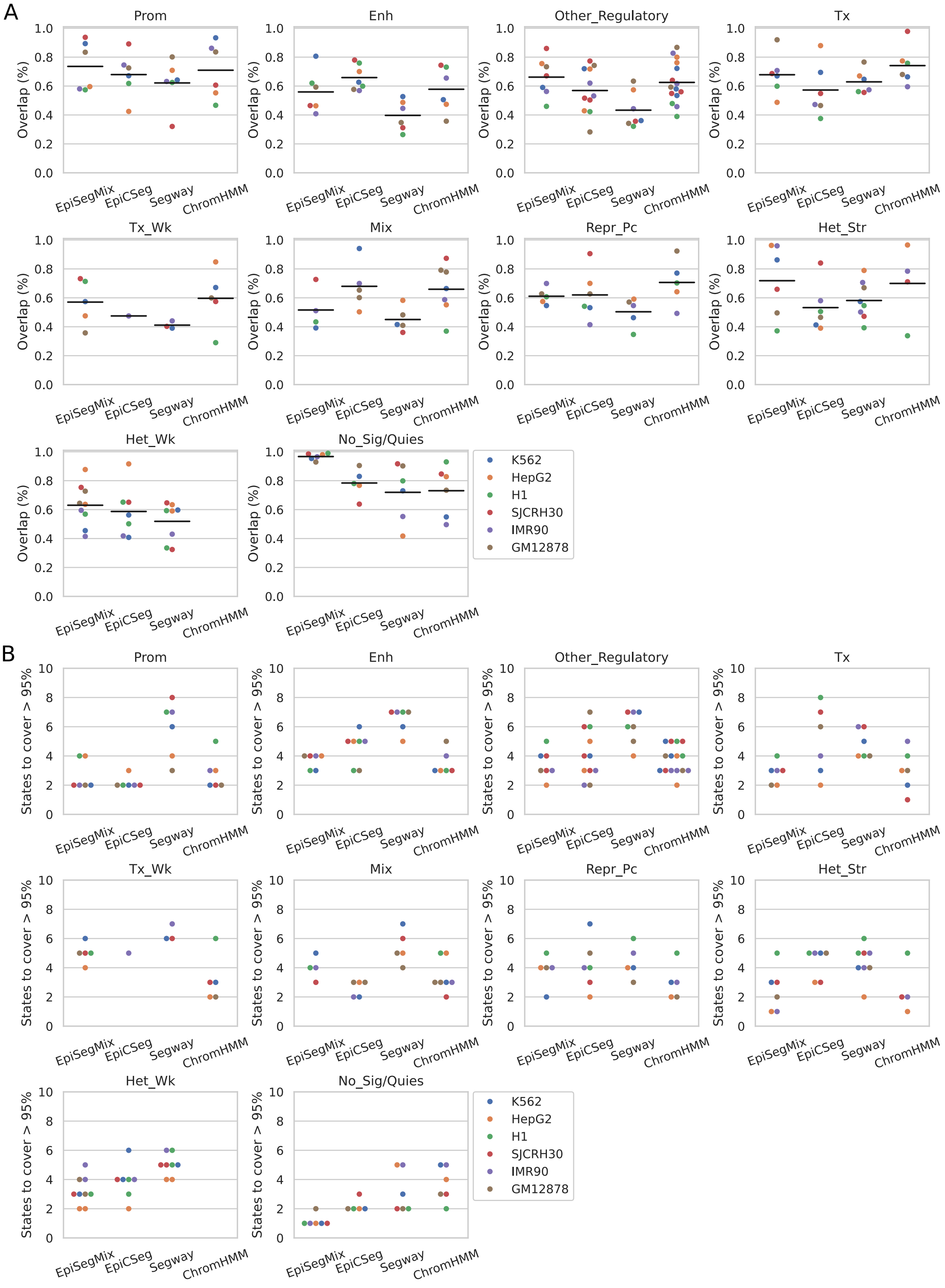

Figure 7: Robustness per state (each state in the first replicate was manually annotated). **A** The percentage of how many base pairs are covered by each state in the second replicate was calculated. Each dot corresponds to the percentage covered by the state with the highest overlap. **B** Number of states of the second replicate that are required such that over 95% of the state in the first replicate are covered.

S7 Supplementary Figure K562

The following 3 figures provide an in-depth look at the results for cell line K562. Figure 8A displays two genomic regions of distinct transcription activity; Figure 8B shows the enrichment patterns of each histone mark across HMM states for each method. Figure 9 visualizes state enhancement near validated enhancers. Figure 10 shows gene activity in regions that ChromHMM classified as weak transcription (Tx_Wk; panel A) or quiescent (Quies; panel B), grouped by the predicted EpiSegMix state. EpiSegMix estimates slightly different acitivity levels per state.

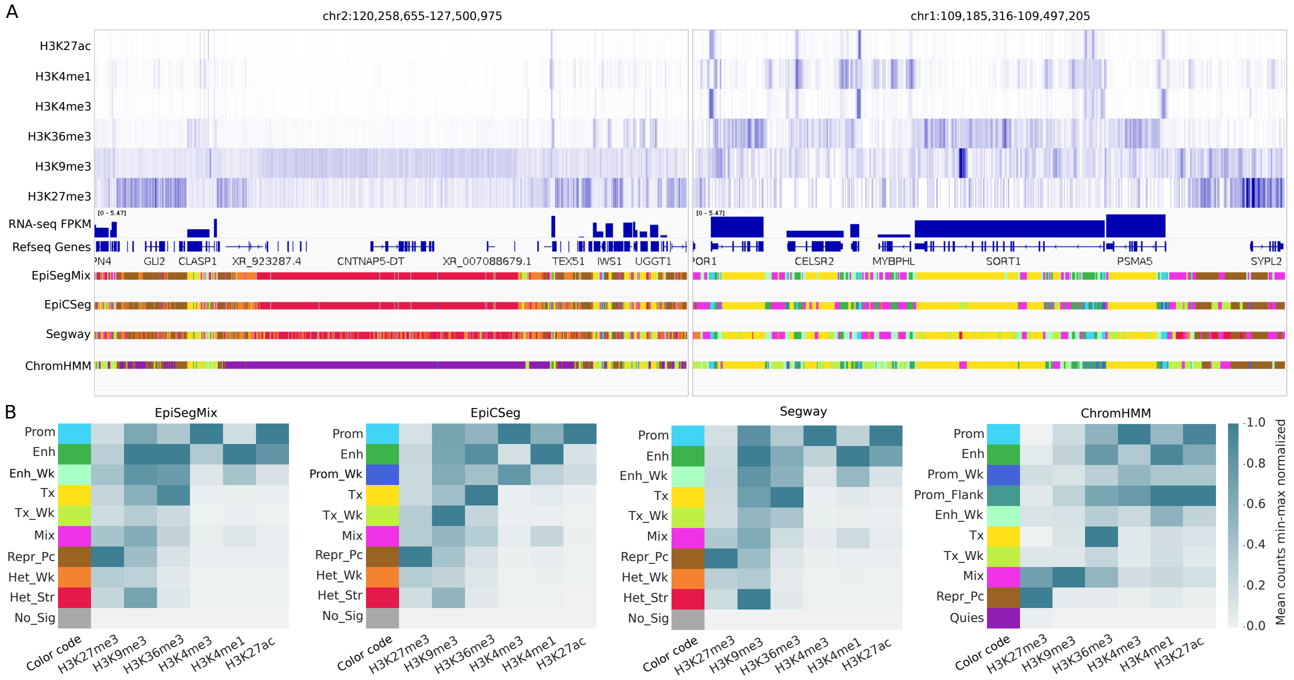

Figure 8: IGV-screenshot for two regions with different genomic activities. **A** The first six tracks show the ChIP-seq intensities of the six input histone marks. The 7th track shows the signal of unique reads from an RNA-seq experiment, and the 8-th track shows the Refseq gene positions. The last four tracks contain the segmentation of EpiSegMix, EpiCSeg, Segway and ChromHMM. On the left, a heterochromatic region is shown which highlights the smooth transition from strong heterochromatin (high H3K9me3) to Polycomb repressed heterochromatin (high H3K27me3). On the right, an actively transcribed region is shown. **B** Heatmap showing the histone patterns in the discovered chromatin states with color legend.

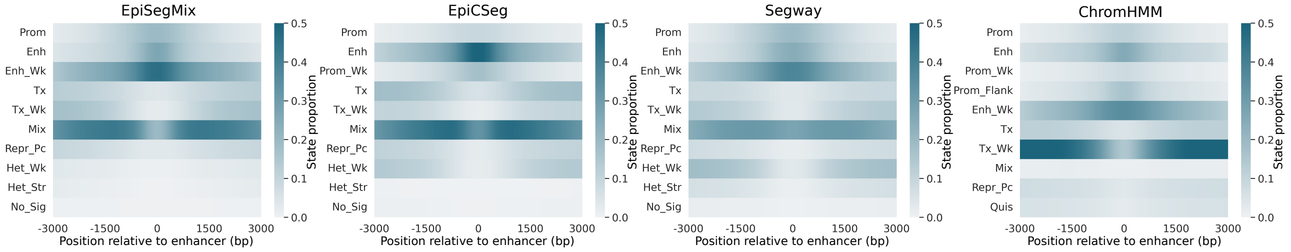

Figure 9: State enrichment near enhancers in K562. Validated enhancers were downloaded from http://enhanceratlas.org/ (Gao et al., 2016) and the state annotation 3000 bp up- and downstream was evaluated. The heatmap shows for each position the percentage of each state.

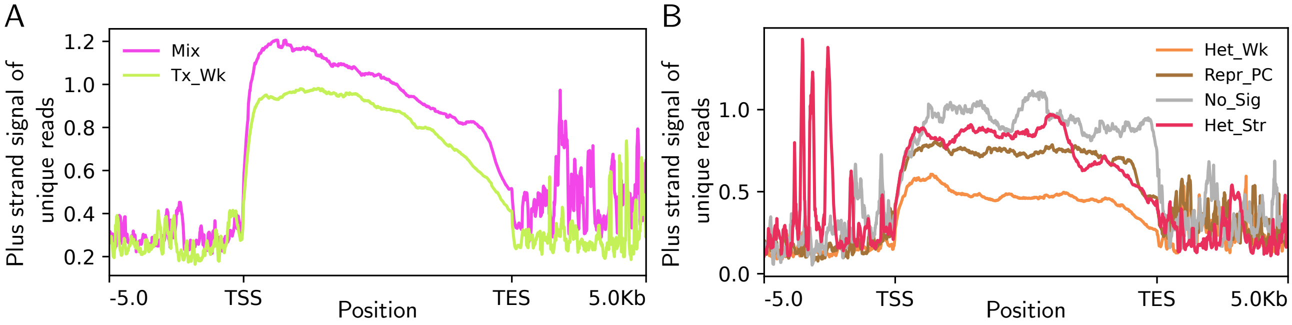

Figure 10: ChromHMM reclassified genes in EpiSegMix. The figure shows the reallocated genes of ChromHMM's Quies state and Tx_Wk states to EpiSegMix well defined states that reflect distinct gene activity. Each gene was annotated with maximum overlapping (in base pairs) states across different methods. All genes were stretched or squished to 10 Kbp along 5 Kbp up and downstream. **A** The protein coding genes that overlapped with Tx_Wk state in ChromHMM and Mix or T_Wk in EpiSegMix were used to plot the gene activity across and around genes using *deeptools* (Fidel et al., 2016). **B** Similar to Figure A, here showing the transition of genes from ChromHMM's Quies to EpiSegMix heterochromatic states.

S8 Biological Annotation of Chromatin States

We provide names, abbreviations and descriptions of the biological names (labels) of the HMM states.

Name	Abbreviation	Description
Promoter	Prom	Promoter state characterized by an enrichment of H3K4me3. Mainly found near transcription start sites.
Enhancer	Enh	Enhancer state showing an enrichment of H3K4me1 (and H3K36me3 in intergenic enhancers).
Enhancer Weak	Enh_Wk	Same as enhancer state but with lower signal intensities (often flanking enhancer state).
Promoter Weak	Prom_Wk	Similar enrichment to promoter state but with lower signal intensities.
Promoter Flanking	Prom_Flank	Low enrichment of promoter-associated marks and mainly flanking promoter regions.
Transcription	Tx	State labeling actively transcribed genes (enriched in gene bodies) and showing high signal intensities of the transcription elongation mark H3K36me3.
Transcription Weak	Tx_Wk	Similar to transcription state but with lower intensities of H3K36me3.
Mix	Mix	State showing an enrichment of several active marks (transcription, regulatory...) and a low intensity of repressive marks, found as intermediate/border state in active regions.
Repressive Polycomb	Repr_Pc	Polycomb repressed heterochromatin, characterized by high levels of H3K27me3. Important for cell-type specific gene repression.
Heterochromatin Strong	Het_Str	State showing an enrichment of the broad heterochromatic mark H3K9me3 (and H3K27me3).
Heterochromatin Weak	Het_Wk	Similar to strong heterochromatin, but with lower signal intensities.
Quiescent	Quies	Low signal intensity for any mark.
No Signal	No_Sig	No signal for any mark (mainly unmappable regions).
Regulatory	Other_Regulatory	Label for all states that show a mixed enrichment of regulatory marks (H3K27ac, H3K4me1, H3K4me3) and are not the main promoter or enhancer state with strongest enrichment of the respective histone modifications.

S9 Data Availability

We provide ENCODE accession numbers to all datasets used in the main article.

S9.1 Histone ChIP-seq Data

Cell line	Replicate	Mark	Accession Bam	Accession bigWig
K562	1	H3K9me3	ENCFF104THG	ENCFF103IHG
K562	1	H3K27me3	ENCFF392ZKG	ENCFF585RVP
K562	1	H3K36me3	ENCFF880HKV	ENCFF782UNG
K562	1	H3K27ac	ENCFF121RHF	ENCFF977KGH
K562	1	H3K4me1	ENCFF352HXD	ENCFF490RQD
K562	1	H3K4me3	ENCFF564SVK	ENCFF545JLB
K562	2	H3K9me3	ENCFF155UQU	ENCFF600IBI
K562	2	H3K27me3	ENCFF905CZD	ENCFF679YZC
K562	2	H3K36me3	ENCFF272JVI	ENCFF860PQP
K562	2	H3K27ac	ENCFF907MNY	ENCFF745TSK
K562	2	H3K4me1	ENCFF415GHS	ENCFF149FZG
K562	2	H3K4me3	ENCFF685PPQ	ENCFF617VRS
HepG2	1	H3K9me3	ENCFF828OFD	ENCFF290MPO
HepG2	1	H3K27me3	ENCFF528YMD	ENCFF740NOD
HepG2	1	H3K36me3	ENCFF956WWT	ENCFF722OKM
HepG2	1	H3K27ac	ENCFF157AQL	ENCFF401PIF
HepG2	1	H3K4me1	ENCFF176CMB	ENCFF432CLC
HepG2	1	H3K4me3	ENCFF203YRQ	ENCFF785EQB
HepG2	2	H3K9me3	ENCFF044CBD	ENCFF170MMA
HepG2	2	H3K27me3	ENCFF962CMS	ENCFF914RTP
HepG2	2	H3K36me3	ENCFF522BKQ	ENCFF145XAZ
HepG2	2	H3K27ac	ENCFF278LPJ	ENCFF153WDH
HepG2	2	H3K4me1	ENCFF678ZFC	ENCFF127FEU
HepG2	2	H3K4me3	ENCFF769SZI	ENCFF053XDJ
GM12878	1	H3K9me3	ENCFF306ENK	ENCFF698SKV
GM12878	1	H3K27me3	ENCFF633BHN	ENCFF119CAV
GM12878	1	H3K36me3	ENCFF353YPB	ENCFF269OIU
GM12878	1	H3K27ac	ENCFF269GKF	ENCFF458CRP
GM12878	1	H3K4me1	ENCFF047NLO	ENCFF785YET
GM12878	1	H3K4me3	ENCFF843BWY	ENCFF346GYG
GM12878	2	H3K9me3	ENCFF889UPU	ENCFF856REE
GM12878	2	H3K27me3	ENCFF565DCK	ENCFF736CNQ
GM12878	2	H3K36me3	ENCFF677MAG	ENCFF831NQV
GM12878	2	H3K27ac	ENCFF201OHW	ENCFF716VWO
GM12878	2	H3K4me1	ENCFF385FLM	ENCFF721OOX
GM12878	2	H3K4me3	ENCFF126HII	ENCFF874RRW
IMR90	1	H3K9me3	ENCFF078EHX	ENCFF610PWA
IMR90	1	H3K27me3	ENCFF547BFL	ENCFF158HZL
IMR90	1	H3K36me3	ENCFF994SJM	ENCFF026EYU
IMR90	1	H3K27ac	ENCFF064XTS	ENCFF891IME
IMR90	1	H3K4me1	ENCFF812CWF	ENCFF051ZVY
IMR90	1	H3K4me3	ENCFF021CLP	ENCFF787RGJ

Cell line	Replicate	Mark	Accession Bam	Accession bigWig
IMR90	2	H3K9me3	ENCFF942QOQ	ENCFF397LLZ
IMR90	2	H3K27me3	ENCFF084CZB	ENCFF795SSM
IMR90	2	H3K36me3	ENCFF197BAZ	ENCFF183VQX
IMR90	2	H3K27ac	ENCFF827KCG	ENCFF847YQV
IMR90	2	H3K4me1	ENCFF927WHP	ENCFF256VMH
IMR90	2	H3K4me3	ENCFF355WLE	ENCFF127NCC
H1	1	H3K9me3	ENCFF898JWE	ENCFF700DAR
H1	1	H3K27me3	ENCFF582IQY	ENCFF417VQQ
H1	1	H3K36me3	ENCFF648ZLN	ENCFF992PQD
H1	1	H3K27ac	ENCFF860ABR	ENCFF038XPO
H1	1	H3K4me1	ENCFF834WMC	ENCFF462RVF
H1	1	H3K4me3	ENCFF638NIJ	ENCFF138BOI
H1	2	H3K9me3	ENCFF963LFX	ENCFF119MXN
H1	2	H3K27me3	ENCFF781BZF	ENCFF780FNS
H1	2	H3K36me3	ENCFF267VHA	ENCFF680DCC
H1	2	H3K27ac	ENCFF693IFG	ENCFF779NOX
H1	2	H3K4me1	ENCFF056UIJ	ENCFF710HLL
H1	2	H3K4me3	ENCFF940GKD	ENCFF438GVD
SJCRH30	1	H3K9me3	ENCFF323SJK	ENCFF900DBA
SJCRH30	1	H3K27me3	ENCFF635JMF	ENCFF990LBZ
SJCRH30	1	H3K36me3	ENCFF522KPA	ENCFF247FAZ
SJCRH30	1	H3K27ac	ENCFF743FZM	ENCFF005XLH
SJCRH30	1	H3K4me1	ENCFF616LJP	ENCFF194LBO
SJCRH30	1	H3K4me3	ENCFF587NXR	ENCFF807NST
SJCRH30	2	H3K9me3	ENCFF278GKD	ENCFF391PQN
SJCRH30	2	H3K27me3	ENCFF003JSI	ENCFF851TAD
SJCRH30	2	H3K36me3	ENCFF514BRD	ENCFF960WMP
SJCRH30	2	H3K27ac	ENCFF721URQ	ENCFF933LDZ
SJCRH30	2	H3K4me1	ENCFF657XIL	ENCFF250THY
SJCRH30	2	H3K4me3	ENCFF984RGN	ENCFF686OMM

S9.2 RNA-seq Data

Cell line	Replicate	Accession
K562	1	ENCFF068NRZ
K562	2	ENCFF928YLB
HepG2	1	ENCFF355TDA
HepG2	2	ENCFF073RKC
GM12878	1	ENCFF678BLG
GM12878	2	ENCFF897XES
IMR90	1	ENCFF958WFW
IMR90	2	ENCFF631BAG
H1	1	ENCFF243GKW
H1	2	ENCFF432RPO
SJCRH30	1	ENCFF771NMO
SJCRH30	2	ENCFF955BPJ

S9.3 ATAC-seq Data

Cell line	Accession
K562	ENCFF512VEZ
HepG2	ENCFF990VCP
GM12878	ENCFF981FXV
IMR90	ENCFF848XMR

References

Bernoulli, J. (1713). *Ars Conjectandi.* impensis Thurnisiorum, fratrum, Basel, Switzerland.

Bilmes, J. (1998). A gentle tutorial of the em algorithm and its application to parameter estimation for Gaussian mixture and hidden Markov models. *CTIT technical reports series.*

Brun, R. and Rademakers, F. (1997). ROOT – An object oriented data analysis framework. *Nuclear Instruments and Methods in Physics Research Section A: Accelerators, Spectrometers, Detectors and Associated Equipment*, 389(1):81–86.

Ewels, P., Magnusson, M., Lundin, S., and Käller, M. (2016). MultiQC: summarize analysis results for multiple tools and samples in a single report. *Bioinformatics*, 32(19):3047–3048.

Fidel, R., Ryan, D. P., Grüning, B., Bhardwaj, V., Kilpert, F., Richter, A. S., Heyne, S., Dündar, F., and Manke, T. (2016). deeptools2: A next generation web server for deep-sequencing data analysis. *Nucleic Acids Research.*

Fisher, R. A. (1941). The negative binomial distribution. *Annals of Eugenics*, 11(1):182–187.

Gao, T., He, B., Liu, S., Zhu, H., Tan, K., and Qian, J. (2016). EnhancerAtlas: a resource for enhancer annotation and analysis in 105 human cell/tissue types. *Bioinformatics*, 32(23):3543–3551.

Griffiths, D. A. (1973). Maximum Likelihood Estimation for the Beta-Binomial Distribution and an Application to the Household Distribution of the Total Number of Cases of a Disease. *Biometrics*, 29(4):637–648.

Johnson, N. L., Kotz, S., Kemp, A. W., et al. (1993). *Univariate discrete distributions.* Wiley, New York, NY [u.a.], 2nd ed edition.

Lindén, A. and Mäntyniemi, S. (2011). Using the negative binomial distribution to model overdispersion in ecological count data. *Ecology*, 92(7):1414–1421.

Poisson, S. (1837). *Recherches sur la probabilité des jugements en matière criminelle et en matière civile: précédées des règles générales du calcul des probabilités.* Bachelier Paris.

Rabiner, L. (1989). A tutorial on hidden Markov models and selected applications in speech recognition. *Proceedings of the IEEE*, 77(2):257–286.

Rigby, R. A., Stasinopoulos, M. D., Heller, G. Z., and Bastiani, F. D. (2019). *Distributions for Modeling Location, Scale, and Shape: Using GAMLSS in R.* Chapman and Hall/CRC, New York.

Rodríguez-Avi, J., Conde-Sánchez, A., Sáez-Castillo, A. J., and Olmo-Jiménez, M. J. (2007). A new generalization of the Waring distribution. *Computational Statistics & Data Analysis*, 51(12):6138–6150.

Schröder, C. and Rahmann, S. (2017). A hybrid parameter estimation algorithm for beta mixtures and applications to methylation state classification. *Algorithms for Molecular Biology*, 12(1):21.

Sichel, H. S. (1992). Anatomy of the generalized inverse Gaussian-poisson distribution with special applications to bibliometric studies. *Information Processing & Management*, 28(1):5–17.

Stein, G. Z., Zucchini, W., and Juritz, J. M. (1987). Parameter estimation for the Sichel distribution and its multivariate extension. *Journal of the American Statistical Association*, 82(399):938–944.
